# Chitooligosaccharides Prevents the Development of Colitis-Associated Colorectal Cancer by Modulating the Intestinal Microbiota and Mycobiota

**DOI:** 10.3389/fmicb.2019.02101

**Published:** 2019-09-18

**Authors:** Minna Wu, Jianmin Li, Yunying An, Puze Li, Wancheng Xiong, Jinsong Li, Dong Yan, Mingyong Wang, Genshen Zhong

**Affiliations:** ^1^Henan Key Laboratory of Immunology and Targeted Therapy, School of Laboratory Medicine, Xinxiang Medical University, Xinxiang, China; ^2^School of Basic Medical Sciences, Xinxiang Medical University, Xinxiang, China; ^3^Shaanxi Provincial Second People’s Hospital, Xi’an, China; ^4^The First Affiliated Hospital of Xinxiang Medical University, Xinxiang, China

**Keywords:** chitooligosaccharides, colitis-associated colorectal cancer, microbiota, mycobiota, high-throughput sequencing

## Abstract

Gut microbes play a crucial role in the development of colorectal cancer. Chitooligosaccharides (COS), are oligomer that are depolymerized from chitosan and possess a wide range of biological activities. In this study, the effects of COS on colorectal cancer (CRC) development were evaluated using azoxymethane and dextran sulfate sodium (AOM/DSS) induced mouse model of CRC (CACM). In the COS-treated CRC group (CMCOS), COS protected mice from CRC by decreasing the disease activity index, tumor incidences and multiplicity, and the mRNA levels of COX-2, IL-6, TNF-α, IL-1β, IL-10, and IKK-β mRNA in colonic epithelial cells. The results of a cage-exchanged experiment, in which mice from the CACMe and CMCOSe treatments exchanged cages every day to interact with microbes, showed that gut microbes play an important role in preventing CAC by COS. The abundances of fecal bacteria (total bacteria, *Lactobacillus*, *Enterococcus*, *Fusobacterium nucleatum* and butyrate-producing bacteria) were detected by qPCR on the 0th, 1st, 3rd, 6th, 9th, and 10th weekends. Furthermore, microbiota and mycobiota were analyzed by high-throughput sequencing on an Illumina MiSeq PE300 system. COS protected mice from CRC by reversing the imbalance of bacteria and fungi, especially by reducing the abundance of *Escherichia–Shigella*, *Enterococcus*, and *Turicibacter*, and increasing the levels of *Akkermansia*, butyrate-producing bacteria and *Cladosporium*.

## Introduction

Colorectal cancer (CRC) is the third most common cause of cancer death in the world (GLOBOCON, 2012), accounting for approximately 862,000 deaths in 2018 (∼8.98% of total cancer-related deaths, respectively) (World Health Organization, 2018, Fact sheet 297). In China, CRC is one of the 5 most commonly diagnosed cancers and has shown a significant upward trend in age-standardized incidence and mortality rates, accounting for 376,300 new cases and 191,000 deaths in 2015 (Chen et al., 2016). Various factors are considered to be responsible for the development of CRC including inheritance (genetic alterations and family history) and environmental factors (diet, smoking, obesity, low physical activity, sex, ethnicity, etc.) (Dulal and Keku, 2014). Chronic inflammation is believed to promote carcinogenesis, and the risk for colon cancer increases with the duration and anatomic extent of colitis as well as the presence of other inflammatory disorders (Ullman and Itzkowitz, 2011; Brennan and Garrett, 2016). Interestingly, gut microbes can be influenced by these factors, and the involvement of gut microbes in colorectal carcinogenesis is becoming increasing clear due to their role in modulating host metabolism, barrier function, and innate and adaptive immunity (Dulal and Keku, 2014; Gagnière et al., 2016).

The human colon is well known to host a highly diverse and complex microbial community that includes bacteria, fungi, viruses and archaea. Many studies have implicated microbial dysbiosis, a pathological imbalance in the microbial community, in the etiology of CRC (Gagnière et al., 2016). Approximately 100 trillion bacteria of more than 1,000 heterogeneous species exist in colon (Human Microbiome Project Consortium, 2012). Some of which have been identified and are suspected to play a role in colorectal carcinogenesis, such as *Fusobacterium* spp., *Escherichia coli*, and *Bacteroides fragilis* (Arthur et al., 2012; Gur et al., 2015; Brennan and Garrett, 2016; Ramos and Hemann, 2017; Dejea et al., 2018). Bacteria also prevented the development of CRC by producing metabolites (e.g., acetate, propionate, and butyrate) (Louis et al., 2014). Due to their low levels in the gut (0.2%, 66 genera and 184 species), fungi are often ignored in studies of intestinal microbes. Recently, mycobiota have been linked with a number of diseases (e.g., colitis, antibiotic associated diarrhea, inflammatory bowel disease, and peptic ulcers) by interacting with microbiota, modulating immunity and producing mycotoxins (Iliev et al., 2012; Liguori et al., 2016; Li et al., 2017; Sokol et al., 2017). However, alteration of the gut mycobiota in CRC patients has rarely been reported. Luan et al. (2015) analyzed the fungal microbiota of biopsy samples from colorectal adenomas (some advanced cases can further develop into carcinoma) and adjacent tissues by sequencing and observed that fungal diversity was decreased in adenomas, while the size of adenomas and disease stage were closely related to changes in the mycobiota. Gao et al. (2017) observed fungal dysbiosis in colon polyps and CRC, including decreased fungal diversity in polyp patients, an increased Ascomycota/Basidiomycota ratio, and an increased proportion of the opportunistic fungi *Trichosporon* and *Malassezia*, which may favor the progression of CRC. Because of its effects on modulating immunity, producing mycotoxins and interacting with microbiota (Van Raay and Allen-Vercoe, 2017), the role of mycobiota in the development and prevention of CRC should be further investigated.

Chitooligosaccharides (COS) are oligomers that are depolymerized from chitosan. COS have been reported to possess a wide range of biological activities, such as antimicrobial, antioxidant, anti-inflammatory, anti-tumor, immunostimulatory, hypocholesterolemic activities (Liang et al., 2016; Qu and Han, 2016; Liagat and Eltem, 2018). COS have been used to inhibit the growth of various bacteria (e.g., *E. coli*, *Salmonella enteritidis*, and *Listeria monocytogenes*), fungi (e.g., *Trichophyton rubrum*) and virus (e.g., HIV-1) (Artan et al., 2010; Mei et al., 2015; Laokuldilok et al., 2017; Sánchez et al., 2017), and showed beneficial effects on probiotic bacteria together with inhibitory effects on intestinal pathogens (Nurhayati et al., 2016). The concentrations of SCFAs and the abundances of *Lactobacilli* and *Bifidobacteria* were significantly increased in the cecum of mice treated with COS (Pan et al., 2009). COS are also effective against various cancer cells *in vitro*, such as prostate, lung, hepatocellular, gastric and colon cancer cells (Park et al., 2011; Han et al., 2015, 2016; Ryu et al., 2017). However, the relationship between the anti-tumor activities of COS and its effects on bacteria *in vivo* remains unclear.

In this study, COS were administered intragastrically to AOM/DSS-induced CRC mice. The anti-tumor activity of COS toward CRC was evaluated by detecting tumor incidence and multiplicity, calculating the disease activity index (DAI), analyzing pathologic characteristics and quantifying the abundance of inflammation-associated factors/cytokines. The role of microbes in preventing the development of CRC was evaluated by exchanging the cages of CRC and COS-treated CRC mice. The dynamics of total bacteria, *Lactobacillus*, *Enterococcus*, *Fusobacterium nucleatum* and butyrate-producing bacteria were evaluated by quantitative PCR (qPCR). High-throughput sequencing was performed to reveal the structure of microbiota and mycobiota, and to determine the specific functional bacteria and fungi that contributed to the preventative effect of COS on CRC.

## Materials and Methods

### Animals and Reagents

Eight-week-old male C57/BL6 mice (22-24 g) were purchased from Vital River Laboratory Animal Technology Co. Ltd. (Beijing, China). All animals were raised in sterilized cages under controlled conditions (i.e., temperature 23 ± 2°C, humidity 55 ± 5%, and 12 h light/dark cycles) and fed sterilized standard rodent chow food and sterilized water under specific pathogen-free conditions.

AOM was purchased from Sigma-Aldrich (St. Louis, MO, United States) and DSS was purchased from MP Biomedicals (molecular weight: 36−50 kDa, MP Biomedicals, Santa Ana, CA, United States) (Sinder et al., 2016). COS was purchased from Dalian Meilun Biotech Co., Ltd. (molecular weight: <3 kDa, Dalian, China). The QIAamp DNA Stool Mini Kits were purchased from Qiagen (HiCILen, Germany), and the Fecal Occulted Test Kits were purchased from Baso Diagnostics Inc. (Zhuhai, China). AOM was dissolved in normal saline to a final concentration of 0.5 mg/mL. DSS and COS were dissolved in sterile deionized water.

### Experimental Procedures

Forty-eight eight-week-old male C57/BL6 mice were randomly and averagely divided into six treatments: the normal healthy treatment (CK), COS control treatment (COS), AOM/DSS-induced colorectal cancer model mice (CACM), COS-treated CACM mice (CMCOS, 300 mg/kg COS in AOM/DSS-induced CRC), and the exchanged CACM (CACMe) and CMCOS (CMCOSe) groups. The number of replications in each treatment was 8. All mice in each treatment were fed a standard sterile rodent chow diet for 10 weeks. Sterile drinking water was provided to the mice in the CK and COS treatments. For the mice in the CACM, CMCOS, CACMe and CMCOSe treatments, CRC was induced using the AOM/DSS procedure, which was described in our previous study (Wu et al., 2016). The mice were injected intraperitoneally with a single dose (10 mg/kg) of AOM on the first day, and 1 week later, three experimental courses with DSS were performed. The mice were provided drinking water with 2% DSS for a week followed by sterile drinking water for 2 weeks in each course. In the COS, CMCOS and CMCOSe treatments, 300 mg/kg COS was administered intragastrically once a day and six times per week, starting from the first day of the study. The mice in CACMe and CMCOSe treatments were raised in individual cages, respectively. The mice from these two groups exchanged cages each other every day, and shared litters to interchange intestinal microbes.

Animal weights were evaluated and recorded at the end of each week. Disease activity index (DAI) curves were generated to evaluate disease progression, which was based on weight, hematochezia, and stool malformation. As described by Menghini et al. (2017), DAI were performed by an experimentalist blinded to the study. In detail, to obtain a total clinical DAI ranging from 0 (healthy) to 4 (maximal score for DSS-induced colitis), the average score of (a) body weight loss (i.e., 0, none; 1, 1–5%; 2, 5–10%; 3, 10–20%; and 4, >20%), (b) stool consistency (i.e., 0, normal; 2, loose stool; and 4, diarrhea), and (c) bloody stool (i.e., 0, negative; 2, fecal occult blood test positive; and 4, gross bleeding) was calculated for each experimental animal. The presence of blood in stools was tested with Fecal Occulted Test kits (Baso Diagnostics Inc., Zhuhai, China).

Fecal samples were collected at the end of the 0th (the day before the beginning of experiment), 1st, 3rd, 6th, 9th, and 10th weekends and stored at −80°C. All mice were sacrificed at the completion of the experiment at the end of the 10th week. Approximately 1-cm sections of colon were cut and immediately placed in 10% buffered formalin for fixation, and the rest of colons were subsequently dissociated, opened longitudinally, and rinsed with phosphate-buffered saline (pH 7.4). The number of tumors determined under a dissecting microscope. Then, the colon tissues were used to isolate epithelial cells as previously described (Tong et al., 2007). The isolated colonic epithelial cells were stored at −80°C for further study.

### Histopathological Analysis

The fixed colon tissues were embedded in paraffin, sectioned at 3–4 μm and stained with hematoxylin and eosin (H&E). All processed sections were subsequently evaluated by a pathologist in a blinded fashion.

### Analysis of Cytokines in Colonic Epithelial Cells by Quantitative RT-PCR

Total mRNA was extracted from colonic epithelial cells using TRIzol reagent (Takara, Dalian, China), as per manufacturer’s instruction, and reverse transcription was conducted using a Reverse Transcription kit (Takara, Dalian, China). The quantity and quality of cDNA was evaluated by 1% (w/v) agarose gel electrophoresis in 0.5 mg/mL ethidium bromide and by Nano Drop 2000 ultraviolet spectrophotometry. RT-qPCR was performed to assess the fold changes in COX-2, IL-6, TNF-α, IL-1β and IL-10 expression using a Step One Plus System (ABI) and the ΔΔCt method (Bhatt et al., 2015).

### Quantitation of Specific Bacteria by qPCR

Fecal bacterial DNA was extracted using a QIAamp DNA Stool Mini Kit (Qiagen, Hilden, Germany) according to the manufacturer’s instructions. The abundances of total bacteria, *Lactobacillus*, *Enterococcus*, *F. nucleatum* and butyrate-producing bacteria were quantified by qPCR. Target gene copy numbers were determined by comparison to a standard curve and normalized to the total DNA. PCR reactions were performed using a Step One Plus System (ABI). The primer sequences and qPCR amplification protocol are shown in Supplementary Table S1.

### High-Throughput Sequencing and Bioinformatics Analysis

Twenty-four fecal samples from the CK, COS, CACM, CMCOS, CACMe, and CMCOSe groups were randomly collected (*n* = 4) to perform high-throughput sequencing of the 16S and 18S rRNA. Genomic DNA was amplified with the bacterial 16S rRNA gene (V3-V5 region) primers 338F/806R and the 18S rRNA gene primers SSU0817/1196. Primers were linked to Illumina sequencing adapters, and the reverse primers contained a sample barcode. The PCR products were purified, and the concentrations were adjusted for sequencing on an Illumina MiSeq PE300 system (MajorBio Co., Ltd., Shanghai, China).

Raw read sequences were demultiplexed and quality-filtered using QIIME (version 1.8.0) (Caporaso et al., 2012) with the following criteria: (i) the 300-bp reads were truncated at any site receiving an average quality score of <20 over a 50-bp sliding window, discarding the truncated reads that were shorter than 50 bp; (ii) exact barcode matching, two nucleotide mismatches in primer matching, and reads containing ambiguous characters were removed; and (iii) only paired-end reads with overlap longer than 10 bp were assembled according to their overlap sequence. Reads that could not be assembled were discarded.

The UCHIME (Edgar et al., 2011) was used to cluster contigs and remove chimera. The optimized sequences were clustered into operational taxonomic units (OTUs) with 97% similarity using UPARSE (version 7.1)^1^ and aligned with SILVA database^2^ using a confidence threshold of 70% (Amato et al., 2013). The data were analyzed on the free online platform of Majorbio I-Sanger Cloud Platform^3^. The alpha diversity analysis was performed using the QIIME 1.8.0 software (Caporaso et al., 2012). Statistical comparison of diversity indexes among different treatments were made by one-way analysis of variance (ANOVA) using the Statistical Package for the Social Sciences (SPSS, version 19.0, Chicago, IL, United States). An analysis of similarity (ANOSIM) was performed using the QIIME 1.8.0 software to determine whether AOM/DSS, COS and cage-exchanged had significantly different bacterial community compositions. Cluster dendrograms, bar plots, principal component analysis (PCA) and principal-coordinate analysis (PCoA), and heatmaps were created in the R software (version 3.7.0). To assess the effect size of each differentially abundant taxon, a metagenomic biomarker discovery approach was performed using LEfSe (linear discriminant analysis [LDA] coupled with effect size measurement)^4^, which performed a non-parametric Wilcoxon sum-rank test followed by LDA analysis (Segata et al., 2011). The bacteria-fungi network was generated using the CoNet plugin (version 1.0b7) for Cytoscape (version 3.6.0) on the basis of non-parametric Spearman correlation coefficients, with a minimal cutoff threshold of 0.6 (*P* < 0.01, Bonferroni corrected) (Faust et al., 2012). Correlation data for dominant genera (relative abundance > 0.1%) that were detected in microbiota and mycobiota are reported.

The raw data were deposited in the Sequence Read Archive (SRA) under the access numbers SRP143438 and SRP144668.

### Statistical Analysis

SPSS (SPSS 19.0, Chicago, IL, United States) was used to perform data analysis, with the results expressed as the mean ± SD (i.e., standard deviation) for individual experiment. If multiple sets of variables were consistent with homogeneity of variance (Kruskal–Wallis *H* test), one-way analysis of variance (ANOVA) was used to compare multi-group variables. Statistical tests were two-sided, and a *P* < 0.05 was considered significant.

### Ethics Statement

This study was carried out in accordance with the recommendations of the Institute Animal Care and Use Committee of Xinxiang Medical University, China. The experimental protocol for animal studies was reviewed and approved by the Institute Animal Care and Use Committee of Xinxiang Medical University, China.

## Results

### COS Prevented CRC Development in C57/BL6 Mice

**FIGURE 1:**
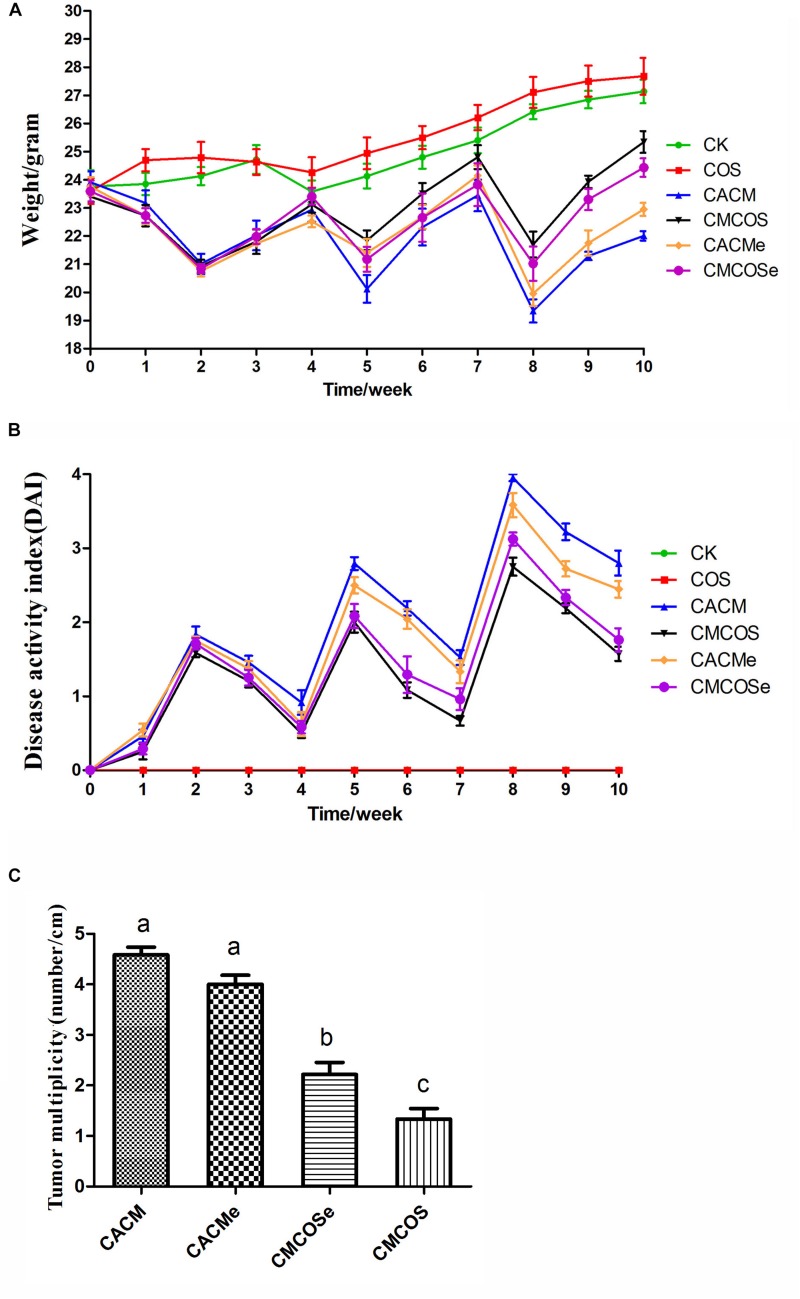
COS protected mouse gastrointestinal tracts from AOM/DSS-induced CAC. **(A)** Changes in body weight. **(B)** DAI values based on weight loss, hematochezia, and diarrhea. **(C)** Tumor multiplicity (number of tumors per cm in colon). The results were presented as the mean ± standard deviation (SD); *n* = 8 for each treatment. Analysis of variance (ANOVA) was used, significant differences (*P* < 0.05) between treatments are indicated by the letters a, b, or c. CK, control treatment; COS, COS control treatment; CACM, AOM/DSS-induced colitis-associated CRC model mice; CMCOS, COS-treated CACM mice (300 mg/kg/d COS); CACMe, exchanged CACM treatment; CMCOSe, exchanged CMCOS treatment.

As shown in Figure 1A, the body weights of the COS group were similar to those of the CK group. Compared with the CK group, significant body weights loss was observed in AOM/DSS-induced CACM treatments (CACM and CACMe), and was effectively rescued by the COS treatment (CMCOS and CMCOSe). Interestingly, the body weight of the CACMe group, which exchanged microbiota with the CMCOSe group, was significantly higher than that of the CACM group, whereas the body weight of the CMCOSe group was obviously lower than that of the CMCOS group at the 10th weekend (*P* < 0.05) (Supplementary Table S2). DAI curves were developed to evaluate disease progression. The DAI scores were 0 in the CK and COS groups throughout the experimental period (Figure 1B). Three peaks corresponding to the three cycles of DSS administration (in drinking water) were observed in the other four groups. The DAI curves of the CACM and CACMe were similar, whereas those of the CMCOS and CMCOSe groups were similar. The COS treatment induced significant decrease of DAI score both in the CMCOS and CMCOSe groups. However, the DAI score in the CACMe was significantly lower than that observed in the CACM group (*P* < 0.05) (Supplementary Table S3).

The histopathological characteristics of tumor tissue samples from each group of mice were evaluated (Supplementary Figure S1 and Supplementary Table S4). The tumor incidences in the CACM, CACMe, CMCOSe, CMCOS, COS, and CK groups were 100, 100, 87.5, 75.0, 0, and 0%, respectively. Tumor multiplicity (the number of tumors per centimeter colon) in the CACM, CACMe, CMCOSe, and CMCOS groups was 4.58 ± 0.38, 4.0 ± 0.45, 2.25 ± 0.52, and 1.58 ± 0.49, respectively (Figure 1C). Tumor multiplicities in the CACM and CACMe groups were significantly higher than that in CMCOSe and CMCOS groups (*P* < 0.05). The COS treatment significantly decreased the tumor incidence and multiplicity; however, the tumor multiplicity in the CMCOSe group was notably higher than that observed in the CMCOS group (*P* < 0.05).

**FIGURE 2:**
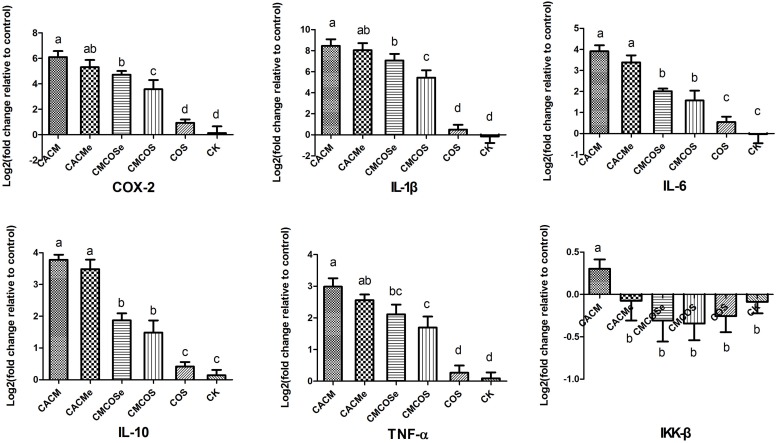
COS inhibited cytokine expression in the CRC mouse at the 10th weekend. The results were presented as the mean ± standard deviation (SD); *n* = 8 for each treatment. Analysis of variance (ANOVA) was used, significant differences (*P* < 0.05) between treatments are indicated by the letters a, b, c, or d. CK, control treatment; COS, COS control treatment; CACM, AOM/DSS-induced colitis-associated CRC model mice; CMCOS, COS-treated CACM mice (300 mg/kg/d COS); CACMe, exchanged CACM treatment; CMCOSe, exchanged CMCOS treatment.

To determine the association between the anti-cancer effects of COS, microbes and cytokine changes in colonic epithelial cells, the abundances of pro-inflammatory factors/cytokines that are often altered in colitis-associated CRC were quantified by RT-qPCR. As shown in Figure 2, the levels of COX-2, IL-1β, IL-6, IL-10 and TNF-α mRNA were significantly increased in the CACM and CACMe groups, whereas the COS treatment significantly reduced the mRNA levels of these genes. It is worth noting that the levels of COX-2 and IL-1β mRNA in the CMCOSe group were significantly higher than those observed in the CMCOS group.

### Dynamic Changes of Specific Bacterial Taxa Based on qPCR

The dynamic changes in the total bacteria and some specific taxa (*Lactobacillus*, *Enterococcus*, *F. nucleatum* and butyrate-producing bacteria) were assessed by qPCR. As shown in Supplementary Figure S2, total bacterial abundances were significantly lower in the CACM group than those observed in the CK and COS groups at the 6th, 9th, and 10th weekends and were significantly higher in the CMCOS group than in the CACM group at the 9th and 10th weekends. The treatment of COS significantly increased the total bacteria abundances at the 9th and 10th weekends in the AOM/DSS-induced CRC diseased mice, whereas there was no significant difference between the CMCOSe and CACMe groups.

After the 3rd weekend, significant differences in the abundance of *F. nucleatum* were observed between healthy mice (CK and COS) and CRC mice (Supplementary Figure S3). At the end of experiment (the 10th weekend), the abundance of *F. nucleatum* was significantly increased in the CACM and CACMe groups but was notably decreased in the COS-treated CACM mice (CMCOS). However, no significant difference was observed between the CMCOSe and CACM groups.

The abundances of the *Enterococcus* genus was significantly increased in the CACM and CACMe groups compared to those observed in the healthy control mice (CK and COS) after the 6th weekend (Supplementary Figure S4). The treatment of COS rescued the increase of *Enterococcus* in the AOM/DSS-induced CRC mice, which was present at significantly lower abundances in the CMCOS group than in the CACM group. However, at the 9th and 10th weekends, the abundance of *Enterococcus* in the CACM group significantly increased compared to that observed in the CACMe group. The abundance of *Enterococcus* also increased along with the development of CRC in the CACM, CACMe, and CMCOSe groups.

As shown in Supplementary Figure S5, the abundance of the *Lactobacillus* genus decreased at the 9th and 10th weekends in the healthy control mice (CK and COS) and in the COS-treated CRC mice (CMCOS and CMCOSe) and was significantly lower than those observed in the CRC mice (CACM and CACMe).

Butyrate-producing bacteria were sensitive to AOM, as this group of bacteria was significantly reduced at the 1st weekend in all groups given an intra-peritoneal injection of AOM (CACM, CACMe, CMCOS, and CMCOSe) (Supplementary Figure S6), with this trend lasted to the end of the experiment. On the 10th weekend, the quantity of butyrate-producing bacteria in the COS-treated CRC mice (CMCOS and CMCOSe) was significantly higher than that observed in the CACM mice (CACM and CACMe). However, the abundance of butyrate-producing bacteria was significantly lower in CMCOSe group than that in the CMCOS group.

### Comparison of Bacterial Community Composition

From the 24 samples, 890,321 valid and trimmed sequences were obtained, with an average sequence length of 442 bp, with 460 OTUs obtained at 97% similarity level. The minimum number of reads (30,008 reads) sub-sample was taken from each sample for subsequent analysis. As shown in Table 1, the observed Coverage values were approximately 1. The alpha diversity indexes, including Shannon, Chao, Ace, and Sob were significantly lower in the CACM and CMCOS than in the CK groups. Compared with the CACM and CACMe groups, the exchange of cages slightly increased alpha diversity, although a significant difference was not observed.

**TABLE 1:** The alpha diversity indexes of microbiota at the 10th weekend determined by high-throughput sequencing.

**Groups**	**Chao**	**Shannon**	**Sobs**	**Ace**	**Simpson**	**Coverage**
CK	392.88 ± 6.30^a^	4.09 ± 0.33^a^	361 ± 12.96^a^	392.35 ± 9.83^a^	0.05 ± 0.03^a^	1.00 ± 0.000276^a^
COS	321.66 ± 25.49^ab^	3.58 ± 0.30^ab^	297 ± 25.47^ab^	326.2 ± 23.52^ab^	0.08 ± 0.04^a^	1.00 ± 0.000291^a^
CACM	228.22 ± 67.49^c^	3.10 ± 0.46^b^	198 ± 63.85^c^	229.02 ± 69.77^c^	0.11 ± 0.05^a^	1.00 ± 0.000415^a^
CMCOS	222.90 ± 49.96^c^	3.03 ± 0.30^b^	191.75 ± 41.71^c^	223.32 ± 48.12^c^	0.10 ± 0.02^a^	1.00 ± 0.000253^a^
CACMe	253.80 ± 36.95^c^	3.10 ± 0.54^b^	211.5 ± 41.00_bc_	241.30 ± 34.83^bc^	0.12 ± 0.07^a^	1.00 ± 0.000247^a^
CMCOSe	260.44 ± 25.93^c^	3.31 ± 0.17^ab^	216.75 ± 21.47^bc^	257.29 ± 24.67^bc^	0.07 ± 0.02^a^	1.00 ± 0.000201^a^

*The results were presented as the mean ± standard deviation (SD); *n* = 4 for each treatment. Analysis of variance (ANOVA) was used, significant differences (*P* < 0.05) between treatments are indicated by the letters a, b, or c. CK, control group (*P* < 0.05); COS, COS-treated group; CACM, AOM/DSS-induced colitis-associated CRC model mice; CMCOS, COS-treated CACM mice (300 mg/kg COS), CACMe, exchanged CACM group; CMCOSe: exchanged CMCOS group.*

**FIGURE 3:**
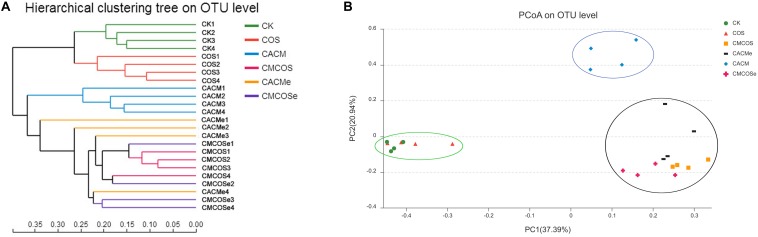
Hierarchical clustering **(A)** and principal co-ordinates analysis (PCoA) of 16S rRNA gene sequences **(B)**. The statistics were performed based on Bray–Curtis distance, *n* = 4. PC1 and PC2 explained 37.39 and 20.94% of variation, respectively. CK, control treatment; COS, COS control treatment; CACM, AOM/DSS-induced colitis-associated CRC model mice; CMCOS, COS-treated CACM mice (300 mg/kg/d COS); CACMe, exchanged CACM treatment; CMCOSe, exchanged CMCOS treatment.

Based on the Bray–Curtis method, the hierarchical clustering with the taxonomic information at the OTU level showed that the bacterial communities in the CK and COS groups clustered into one branch, while all of the AOM/DSS treatment groups were classified into another cluster (Figure 3A). However, the CACM group clustered away from COS-treated CACM groups (CMCOS and CMCOSe) and the exchanged CACM group (CACMe). Similarity, principal co-ordinates analysis (PCoA) also revealed that the fecal bacterial community in the CACM group was different from those present in the other groups (Figure 3B). Samples from the CMCOS, CMCOSe, and CACMe groups were clustered together and were distinct from the healthy (CK and COS groups) and CACM groups. The cluster tree and PCoA indicated that the bacterial communities were separated based on AOM/DSS, COS and the exchange of cages.

The analysis of similarities (ANOSIM) at OTU (*R* = 0.8398, *P* = 0.001), species (*R* = 0.7789, *P* = 0.001), genus (*R* = 0.6866, *P* = 0.001), and family (*R* = 0.6706, *P* = 0.001) levels was conducted. As shown in Supplementary Figure S7, the inter-treatment differences were greater than the intra-treatment differences at the family, genus, species and OTU levels in all of the treatments. These results indicated that there were significant effects of AOM/DSS, COS and cage-exchanged on intestinal bacterial community composition.

**FIGURE 4:**
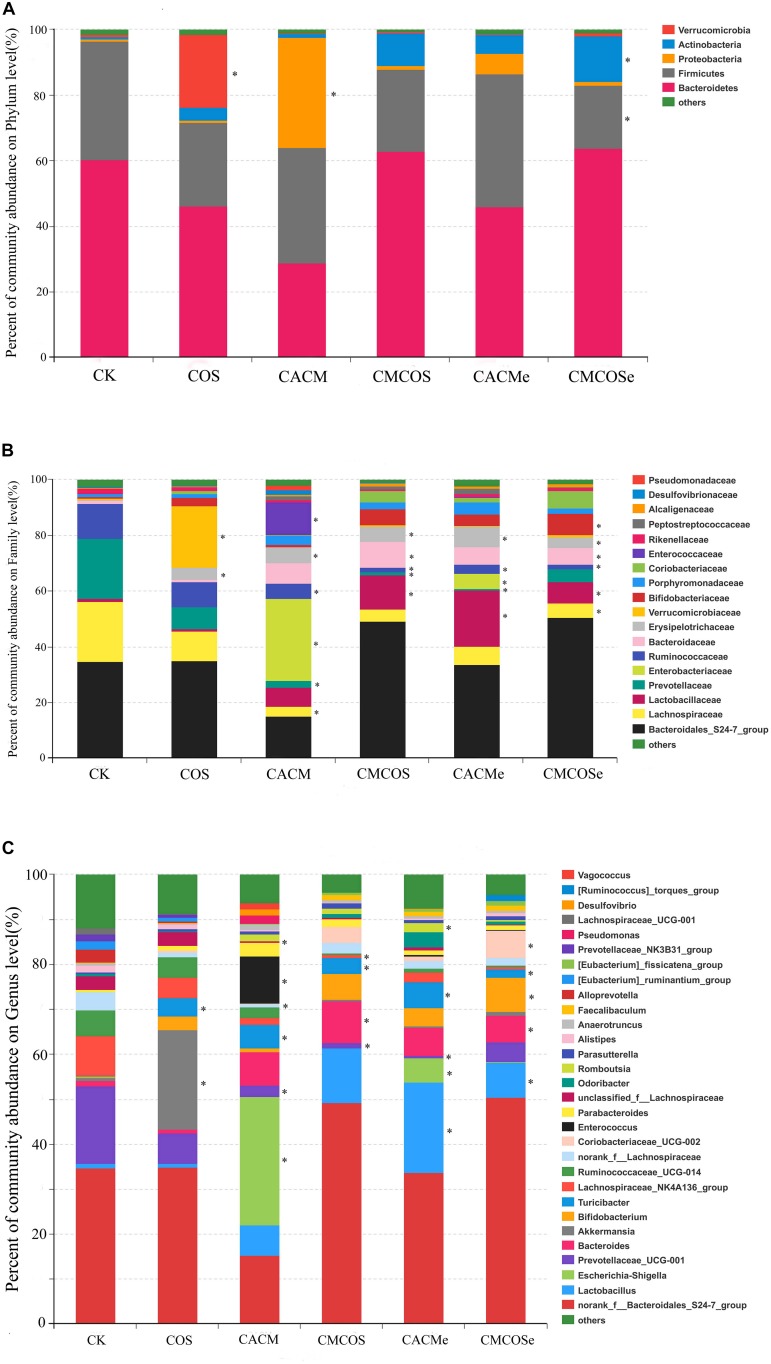
Relative abundances at the phylum **(A)**, family **(B)**, and genus **(C)** levels for bacteria that exceeded 1% of the total. The results are presented as the mean ± standard deviation (SD); *n* = 4 for each treatment. ^∗^Analysis of variance (ANOVA) was used, significant differences between CK and treatments were indicated (*P* < 0.05). CK, control treatment; COS, COS control treatment; CACM, AOM/DSS-induced colitis-associated CRC model mice; CMCOS, COS-treated CACM mice (300 mg/kg/d COS); CACMe, exchanged CACM treatment; CMCOSe, exchanged CMCOS treatment.

To analyze the bacterial composition in detail, the percent of community abundance that exceeded 1% at the phylum, family, and genus levels are present in Figure 4. Based on the taxonomic results, Bacteroidetes and Firmicutes were the most predominant phyla, accounting for 28.89 ∼ 63.70% and 19.26 ∼ 40.71%, respectively (Figure 4A). Compared with the CK group, the percentages of Verrucomicrobia increased in the COS group (22.24 ± 11.60% *vs.* 0.65 ± 0.47%, *P* < 0.05), whereas Proteobacteria increased in the CACM groups (33.61 ± 9.01% *vs.* 0.82 ± 0.19%, *P* < 0.05) and Firmicutes decreased in CMCOSe group (19.26 ± 0.75% *vs.* 36.23 ± 12.87%, *P* < 0.05).

As shown in Figure 4B, 18 families that exceeded 1% of total bacteria in abundance were observed. Compared with the CK group, the COS treatment significantly increased the abundance (%) of Verrucomicrobiaceae (22.24 ± 6.60% *vs.* 0.65 ± 0.17%) and Erysipelotrichaceae (22.24 ± 5.60% *vs.* 0.65 ± 0.47%). In the CACM group, the percentages of Lachnospiraceae, Prevotellaceae and Ruminococcaceae significantly decreased (3.41 ± 1.75% *vs.* 21.62 ± 9.17%, 2.51 ± 1.07% *vs.* 21.56 ± 7.88%, 5.30 ± 2.86%, *vs.* 12.57 ± 4.21%, respectively), whereas those of Enterobacteriaceae, Erysipelotrichaceae, and Enterococcaceae notably increased (29.53 ± 7.05% *vs.* 0.005 ± 0.002%, 11.88 ± 7.50% *vs.* 0.003 ± 0.0009%, 5.74 ± 2.64% *vs.* 0.22 ± 0.09%, respectively). In the COS-treated CACM group, the increases of Enterobacteriaceae and Enterococcaceae were reversed, and no significant difference was observed between the CK and CMCOS groups with respect to Enterobacteriaceae and Enterococcaceae.

At the genus level, 30 genera that exceeded 1% of the total bacteria were observed (Figure 4C). Compared with the CK groups, the COS treatment significantly increased the percentage of *Akkermansia* (22.24 ± 11.60% *vs.* 0.65 ± 0.47%). In the CACM group, the percentages of *Escherichia–Shigella* (28.52 ± 7.06% *vs.* 0.0047 ± 0.0019%), *Enterococcus* (10.57 ± 3.70% *vs.* 0.0028 ± 0.0019%) and *Turicibacter* (5.32 ± 2.90% *vs.* 0.055 ± 0.021%) were significantly increased, whereas those of Prevotellaceae_UCG-001 (2.51 ± 1.07% *vs.* 17.21 ± 10.41%), norank_f_Lachnospiraceae (0.78 ± 0.39% *vs.* 3.95 ± 2.78%) and unclassified_f_Lachnospiraceae (0.44 ± 0.24% *vs.* 3.08 ± 0.89%) were notably decreased (*P* < 0.05). However, the COS-treated evidently reversed the increases of *Escherichia–Shigella* and *Enterococcus* that were induced by AOM and DSS. No significant difference was observed between the CK and CMCOS groups at with respect to *Escherichia–Shigella* and *Enterococcus*, while Prevotellaceae_UCG-001 (1.13 ± 0.76% *vs.* 17.21 ± 10.41%) and Lachnospiraceae_NK4A136_group (0.055 ± 0.021% *vs.* 3.53 ± 1.83%) decreased, *Turicibacter* (3.53 ± 1.83% *vs.* 0.055 ± 0.021%) and Bacteroides (8.84 ± 5.27% *vs.* 0.63 ± 0.26%) significantly increased (*P* < 0.05).

**FIGURE 5:**
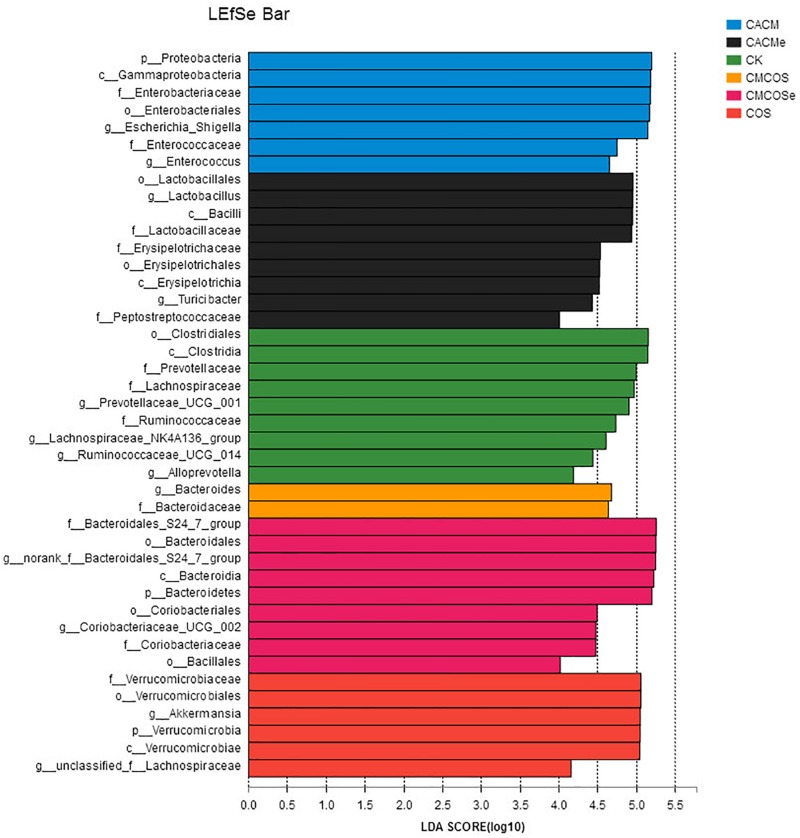
Histogram of the LDA scores for differentially abundant bacteria among the six treatments. Non-parametric factorial Kruskal–Wallis (KW) sum-rank test was used. *n* = 4. CK, control treatment; COS, COS control treatment; CACM, AOM/DSS-induced colitis-associated CRC model mice; CMCOS, COS-treated CACM mice (300 mg/kg/d COS); CACMe, exchanged CACM treatment; CMCOSe: exchanged CMCOS treatment.

LaLEfSe analysis was subsequently performed from phylum to genus level to obtain a cladogram representation and the characteristic bacteria of the gut microbiota for the six treatments (Figure 5). Prevotellacea, Lachnospiraceae, and Ruminococcaceae were dominant in the CK group, whereas *Akkermansia* and Lachnospiraceae in the COS group, *Escherichia_Shigella* and *Enterococcus* in the CACM group, and *Bacteroides* in the CMCOS group. The exchange of cages induced shifts in the microbiota, with characteristic taxa of *Lactobacillus* and *Turicibacter* observed in the CACMe group, whereas Bacteroidales_S24-7_group and Coriobacteriaceae_UCG_002 were observed in the CMCOSe group.

### Comparison of Fungal Community Composition

In total, 876,569 valid and trimmed sequences were obtained from all 24 samples (*n* = 4), with an average of 36,524 sequences per sample and an average length of 401 bp per sequence. All samples were subsampled at same sequence depth (30,452 reads). The total number of OTUs at the 97% similarity level was 117. According to the results of PCA and hcluster, two outliers from the CK and the COS groups were removed in the subsequent analyses, respectively.

The alpha diversity indexes of mycobiota are presented in Table 2. The Chao and Shannon indexes for the CACM group were significantly lower than that observed in the CK group, whereas the Simpson index for the CACM group was significantly higher than that observed in the CK, CMCOS, and CMCOSe groups. AOM and DSS significantly decreased the alpha diversity index values of mycobiota, while the COS treatment increased them. In addition, exchange of cages was less effective at promoting gut fungal diversity.

**TABLE 2:** The alpha diversity indexes of mycobiota at the 10th weekend determined by high-throughput sequencing.

**Groups**	**Chao**	**Shannon**	**Simpson**	**Sobs**	**Ace**	**Coverage**
CK	46.17 ± 9.65^a^	2.59 ± 0.23^a^	0.12 ± 0.04^a^	36.33 ± 7.02^a^	65.71 ± 41.86^a^	1.00 ± 0.000153^a^
COS	33.83 ± 7.91^ab^	2.23 ± 0.32^ab^	0.18 ± 0.06^ab^	30.00 ± 2.65^a^	43.18 ± 22.83^a^	1.00 ± 0.000122^a^
CACM	28.00 ± 4.08^b^	1.78 ± 0.14^b^	0.30 ± 0.01^a^	26.75 ± 4.57^a^	31.29 ± 5.61^a^	1.00 ± 0.000054^a^
CMCOS	35.75 ± 8.50^ab^	2.51 ± 0.35^a^	0.13 ± 0.04^b^	35.50 ± 8.19^a^	37.13 ± 9.90^a^	1.00 ± 0.000065^a^
CACMe	36.08 ± 6.04^ab^	2.22 ± 0.17^ab^	0.21 ± 0.07^ab^	35.00 ± 4.97^a^	36.15 ± 5.87^a^	1.00 ± 0.000048^a^
CMCOSe	40.56 ± 5.03^ab^	2.37 ± 0.29^a^	0.17 ± 0.08^a^	38.25 ± 3.69^a^	41.11 ± 4.74^a^	1.00 ± 0.000082^a^

*The results were presented as the mean ± standard deviation (SD); *n* = 3 for the CK and COS groups, *n* = 4 for the CACM, CMCOS, CACMe, and CMCOSe groups. Analysis of variance (ANOVA) was used, significant differences (*P* < 0.05) between treatments are indicated by the letters a, b, or c. CK, control group; COS, COS-treated group; CACM, AOM/DSS-induced colitis-associated CRC model mice; CMCOS, COS-treated CACM mice (300 mg/kg COS); CACMe, exchanged CACM group; CMCOSe, exchanged CMCOS group.*

The PCA results showed that the samples from the CACM and CACMe groups clustered, while the samples from the other groups clustered together (Supplementary Figure S8). These indicated that the COS treatment reversed the shifts in the fungal communities induced by AOM/DSS. However, the effect of the exchange of cages on the mycobiota was limited.

Based on the taxonomic results, 7 phyla that exceed 1% in abundance were detected (Supplementary Figure S9). Ascomycota and Basidiomycota were the most predominant phyla, accounting for 70.59 ∼ 86.61% and 9.42 ∼ 26.30%, respectively. However, no significant difference was detected between any two groups at the phylum level. Twenty-nine families exceeding 1% in abundance are presented in Supplementary Figure S9, and Trichocomaceae was the most predominant family. No significant difference was observed between the CK and COS groups. Compared with the CK group, norank_o_Sporidiobolales and o_norank_c_Agaricomycetes significantly decreased in the CACM group (0 *vs.* 5.95 ± 1.42%; and 0.11 ± 0.07% *vs.* 4.84 ± 1.34%, respectively). In addition, Cystofilobasidiaceae decreased in the CMCOS group significantly (0.32 ± 0.14% *vs*.4.24 ± 0.75%) (*P* < 0.05). Due to the limitation of the fungal database, only 29 genera exceeding 1% in abundance were detected (Supplementary Figure S9). Unclassified_f_ Trichocomaceae, unclassified_o_Hypocreales, *Boeremia* and *Cladosporium* were the most dominant genera. Between the CK and CACM groups, there were significant differences in norank_o_Sporidiobolales and o_norank_c_Agaricomycetes at the genus level (*P* < 0.05). Compared with the CK, *Mrakia* was significantly decreased in the CMCOS group (0.32 ± 0.14% *vs*.4.24 ± 0.75%) (*P* < 0.05).

**FIGURE 6:**
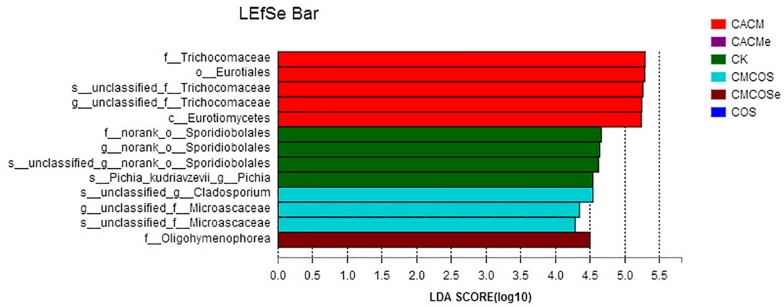
Histogram of the LDA scores for differentially abundant fungi among the six treatments. Non-parametric factorial Kruskal–Wallis (KW) sum-rank test was used. *n* = 3 for the CK and COS groups, *n* = 4 for the CACM, CMCOS, CACMe, and CMCOSe groups. CK, control treatment; COS, COS control treatment; CACM, AOM/DSS-induced colitis-associated CRC model mice; CMCOS, COS-treated CACM mice (300 mg/kg/d COS); CACMe, exchanged CACM treatment; CMCOSe, exchanged CMCOS treatment.

LaLEfSe analysis from the phylum to species level was then performed to obtain a cladogram representation and the characteristic fungi of the gut mycobiota within the six groups. As shown in Figure 6, the greatest differences in taxa among the six groups were detected by LDA (score 3.0). In the CACM group, Trichocomaceae and Eurotiales were the characteristic fungi, while Sporidiobolales and *Pichia* in the CK group, *Cladosporium* and Microascaceae in the CMCOS group, and Oligohymenophorea in the CMCOSe group were the characteristic fungi.

### The Correlation Between Gut Microbes and Environmental Factors

**FIGURE 7:**
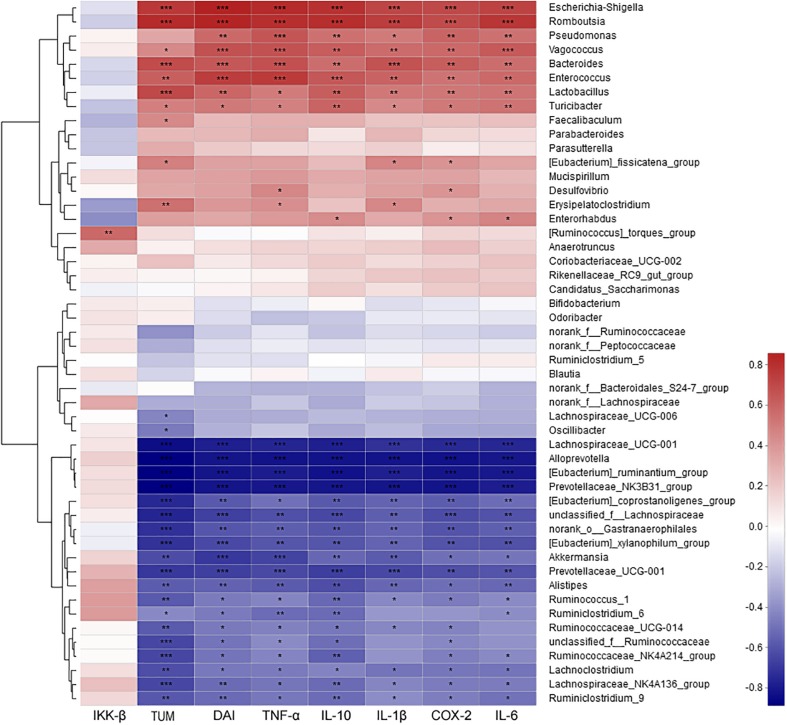
The correlation between microbiota at genus level and environmental factors (DAI, tumor multiplicity and cytokines). The most abundant 50 genera in each sample were used to perform the hierarchical clustering and heatmap analyses based on Spearman correlation coefficient. *n* = 4. TUM, tumor multiplicity; DAI, disease activity index.

The correlation between intestinal microbes (the most abundant 50 genera or species) and environmental factors (DAI, tumor multiplicity and cytokines), were analyzed by Spearman correlation coefficient based on hierarchical clustering and heatmap analyses. At the genus level, some genera were strongly positively correlated with disease traits and cytokine mRNA abundances, such as *Escherichia–Shigella*, *Romboutsia*, *Pseudomonas*, *Vagococcus*, *Bacteroides*, *Enterococcus*, *Lactobacillus*, and *Turicibacter*. Some taxa were strongly negatively correlated with disease traits and cytokines, such as Lachnospiraceae_UCG-001, *Alloprevotella*, *Ruminantium* group, Prevotellaceae_NK3B31 group, *Akkermansia*, and *Alistipes* (Figure 7). Further analysis at the species level showed that most of highly correlated bacteria were unclassified species from the corresponding genera at the genus level. However, some detailed species also were obtained, with *Bacteroides uniformis*, *Bacteroides caccae*, *Lactobacillus johnsonii*, and *Vagococcus fluvialis* were strongly positively correlated with disease severity (Supplementary Figure S10).

**FIGURE 8:**
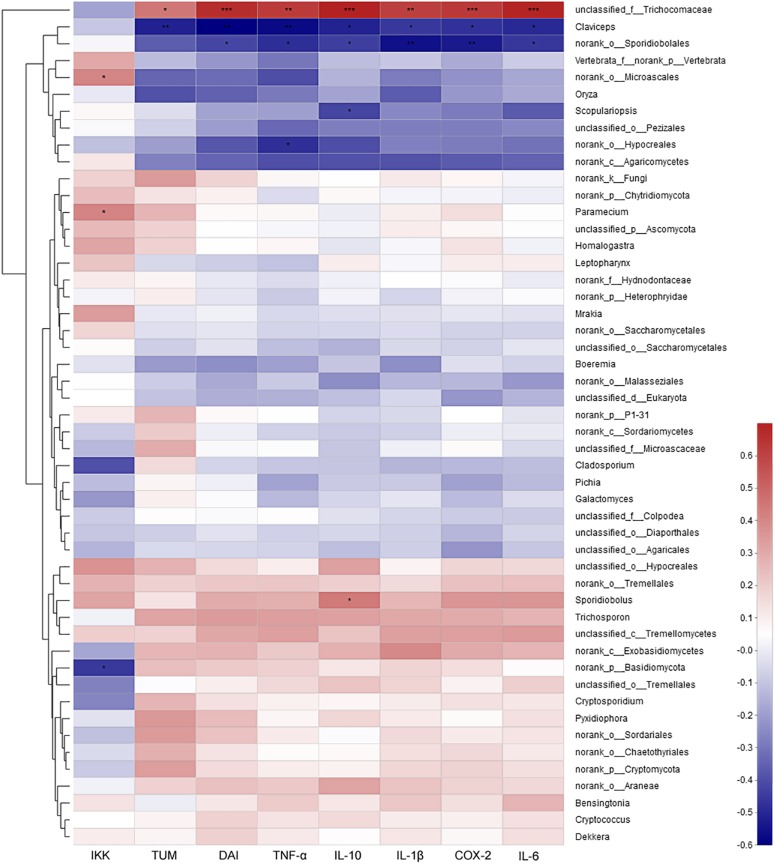
The correlation between mycobiota at genus level and environmental factors (DAI, tumor multiplicity and cytokines). The most abundant 50 genera in each sample were used to perform the hierarchical clustering and heatmap analyses based on Spearman correlation coefficient. TUM, tumor multiplicity; DAI, disease activity index.

The correlation analysis between mycobiota and the assayed factors of disease severity (DAI, tumor multiplicity and cytokines) showed that only several fungi were involved. At the genus level, unclassified Trichocomaceae was positively correlated, while *Claviceps* and norank of Sporidiobolales were strongly negatively correlated with severely these factors (Figure 8). At the species level, *Pichia Kudriavzevii*, *Cladosporium herbarum* and unclassified Sporidiobolales were the most negatively correlated species, while unclassified Trichocomaceae and unclassified Basidiomycota were the most positively correlated species (Supplementary Figure S11).

### The Co-association of Bacteria and Fungi

**FIGURE 9:**
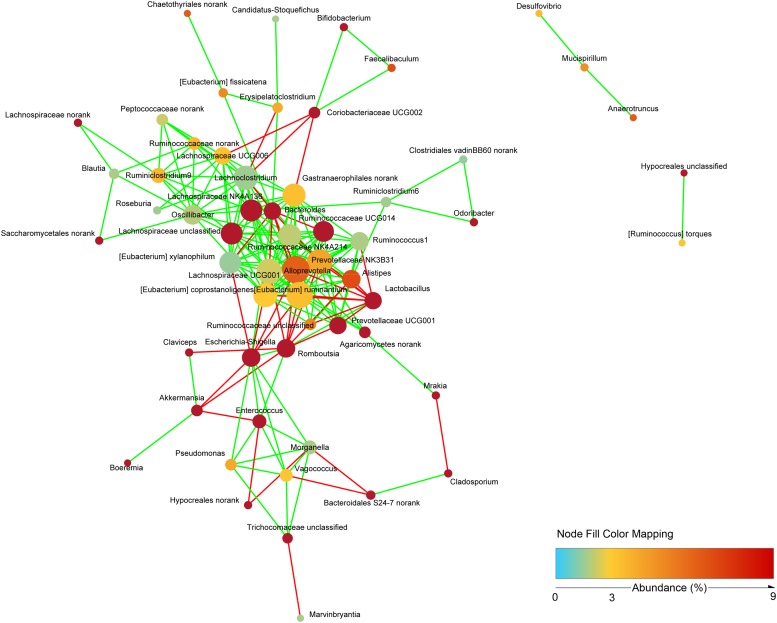
Network analysis indicating interactions among dominant bacteria and fungi. The dominant bacterial and fungal genera that relative abundance >0.1% were used to generated network based on non-parametric Spearman correlation coefficients.

To study the possible interactions among microbiota and mycobiota, the dominant genera (relative abundance > 0.1%) were used to construct a network. Topological properties are always used in network analysis to describe the complex pattern of interrelationships. As shown in Figure 9, the network included 58 nodes and 201 edges. The average network distance between all pairs of nodes (average path length) was 2.97. The clustering coefficient (the degree to which they tend to cluster together) was 0.51. The heterogeneity value (the possibility of hubs) of this network was 0.792. The majority of the network generated was cooccurrent interactions (green lines), whereas the others exhibited mutual exclusions (red lines). *Alloprevotella* (19 edges), *Eubacterium ruminantium* (18 edges), Lachnospiraceae UCG001 (17 edges) and Prevotellaceae NK3B31 (17 edges) were the key genera of microbiota; and norank Agaricomycetes (5 edges) and unclassified Trichocomaceae (4 edges) were the key genera of mycobiota. However, few interactions were observed between bacteria and fungi.

## Discussion

Chitooligosaccharides are considered to be potential anticancer agents because of their anti-tumor activities. In this study, the treatment of COS notably decreased the DAI, the incidence and multiplicity of tumors in the CMCOS mice. These results indicated that COS suppress the development of CRC induced by AOM/DSS. In the COS-treated CRC mice, the levels of COX-2, IL-1β, IL-6, IL-10, and TNF-α mRNA decreased significantly (*P* < 0.05). These results are consistent with previous studies that showed the suppressive effects of gallate-COS on COX-2 mRNA transcription and TNF-α protein expression in A549 human lung epithelial cells (Vo et al., 2017) and NF-κB mediated inflammatory related protein expression in CRC mice (Mattaveewong et al., 2016).

Host genetic and environmental factors, such as (but not limited to) food, bedding, caging, and temperature shape the gut microbiota and account for their variability (Campbell et al., 2012; McCoy et al., 2017). However, litter and cohabitation had a detectable and measurable contribution in the variation of the gut microbial communities in the same mouse genetic background mouse, as mice housed in the same cage begin to share similar gut microbiota due to mixing by coprophagia (Benson et al., 2010; Campbell et al., 2012; Buffington et al., 2016). In this study, to protect the mice with more severe disease in the CACMe group than those in the CMCOSe, the cages of the mice were exchanged (shared litters) rather than cohousing. Strikingly, the DAI score, tumor multiplicity and IKK-β mRNA abundance were significantly decreased in the CACMe group compared to the CACM group, whereas the tumor multiplicity and the COX-2 and IL-1β mRNA abundance were significantly increased in the CMCOSe group compared to the CMCOS group. These results indicated that the cage-exchanged between the CACM and CMCOS mice effectively impacted the development of CRC. Compared with the independently caged CACM or CMCOS groups, the microbial community structure was shifted in the corresponding group with exchanged cages (CACMe or CMCOSe). At the end of the 9th and 10th weekends, compared with the CMCOS group, the abundance of *Enterococcus* and butyrate-producing bacteria in the CMCOSe group was significantly increased and decreased, respectively. The samples from the CACMe group clustered away from those of the CACM group but were close to those of the CMCOSe and CMCOS groups in the PCoA analysis of microbiota. The values of fungal diversity indexes (Chao, Simpson, and Shannon) in the CACM group were different from those in the CK group; however, the cage-exchanged reversed these alterations. Furthermore, LEfSe analysis showed that the characteristic bacteria and fungi shifted in the CACMe (compared to CACM) and CMCOSe (compared to CMCOS) groups. The correlation analysis between environmental factors and intestinal microbes also showed the high degree of correspondence with LEfSe. Thus, the daily exchange of cages effectively promoted the interchange of microbiota and mycobiota between the CACMe and CMCOSe groups, indicating that intestinal microbes (especially microbiota) play an important role in the development of CRC and the prevention of COS on CAC.

Consistent with previous studies, the imbalances of microbiota were observed in the AOM/DSS-induce CRC mice, including decreases in diversity, the reduction of total bacteria and butyrate-producing bacteria, the enrichment of *Enterococcus* and *Escherichia–Shigella*. However, in this study, the treatment of COS alleviated these imbalances and inhibited CRC development. Specific gut bacteria (*Fusobacterium nucleatum*, *Bacteroides fragilis*, *Enterococcus faecalis*, *Escherichia coli*, etc.) are well known to contribute to the development of CRC through a number of potential mechanisms, including promoting chronic inflammation, DNA damage and the production of bioactive carcinogenic metabolites (Keku et al., 2015). Short chain fatty acids (SCFAs) are generally considered to be beneficial metabolites in the human gut, and butyrate in particular is known to have anti-inflammatory effects through histone deacetylase inhibition and subsequent downregulation of proinflammatory cytokines (Chang et al., 2014). Prebiotics and traditional Chinese medicines reduce the risk of colitis and CRC development by influencing the microbiota. The result of a study by Higashimura et al. (2016) suggested that the oral intake of agaro-oligosaccharides prevents high-fat diet-induced gut dysbiosis, thereby inhibiting colon carcinogenesis. In another study, black raspberry anthocyanins played a central role in the chemoprevention of CRC by changing inflammation and the methylation status of the SFRP2 gene and modulating the composition of gut commensal microbiota (the abundance of *Desulfovibrio* sp. and *Enterococcus* spp. were decreased significantly, whereas probiotics such as *Eubacterium rectale*, *Faecalibacterium prausnitzii*, and *Lactobacillus* were dramatically increased) (Chen et al., 2018). Huangqin decoction ameliorates DSS-induced colitis by altering of the gut microbiota (Yang et al., 2017). The results of our previous study also suggested that isoliquiritigenin protects mice from AOM/DSS-induced CRC by reducing the abundances of pathobionts (*Escherichia* and *Enterococcus*) and increasing those of probiotics, particularly butyrate-producing bacteria (*Butyricicoccus*, *Clostridium*, and *Ruminococcus*).

Of the genera altered by the AOM/DSS treatment, the genus *Turicibacter* is of particular interest. The intestinal *Turicibacter* population, which was previous shown to be a strong indicator of wild-type mice, exhibited low competitiveness and was unable to persist in the altered gut resulting from CD45 inactivation (Dimitriu et al., 2013). Zenewicz et al. (2013) also demonstrated a reduction in the abundance of *Turicibacter* in the GI of IL-22 deficiency mice. However, in this study, compared with the CK and COS groups, the abundance of *Turicibacter* significantly increased in all of the AOM/DSS-treated groups, including the CACM, CACMe, CMCOS and CMCOSe groups. An increased abundance of *Turicibacter* was also observed in our previous studied mice with AOM/DSS-induced CRC, where isoliquiritigenin treatment was shown to decrease the abundance of *Turicibacter* in CRC mice (Wu et al., 2016). Munyaka et al. (2016) investigated the fecal and colonic mucosa microbial composition and functional changes in mice treated with DSS and found that *Turicibacter* was positively correlated with the DSS-treated group but negatively correlated with the control group in the fecal and colonic samples. In light of these data, *Turicibacter* may simply be sensitive to DSS and increase in abundance during or after the development of colitis or CRC. Thus, the role of *Turicibacter* in CRC development needs to be further studied.

It is important to note that the abundance of *Akkermansia* was significantly higher in the COS group than in any other groups. Recently, the results of increasing numbers of studies have indicated that *Akkermansia muciniphila* is a component of the healthy gut microbiome and is a potential probiotic. *A. muciniphila* is positively correlated with a lean phenotype, reduced body weight gain, amelioration of metabolic responses and the restoration of gut barrier function by modulation of mucus layer thickness (Dao et al., 2016; Ottman et al., 2017). The Amuc_1100 protein, which was purified from the outer membrane of *A. muciniphila*, induced production of specific cytokines through activation of Toll-like receptor (TLR) 2 and TLR4, improved the gut barrier and partly recapitulates the beneficial effects of the bacterium (Ottman et al., 2017; Plovier et al., 2017). *Akkermansia* also has a positive role in inhibiting cancer development. The clinical responses of cancer patients to immune checkpoint inhibitors were closely correlated with the relative abundance of *A. muciniphila*, and oral supplementation with *A. muciniphila* after fecal microbiota transplantation with non-responder feces restored the efficacy of the PD-1 blockade in an interleukin-12-dependent manner by increasing the recruitment of CCR9^+^CXCR3^+^CD4^+^ T lymphocytes into mouse tumor beds (Routy et al., 2018). HuR inhibition in APC ^*Min*^ mice, a model of FAP and colon cancer, decreased the number of small intestinal tumors, and increased the abundance of *Prevotella, Akkermansia*, and *Lachnospiraceae* (Lang et al., 2017). The dramatic increase of *Akkermansia* in the COS group in this study suggested that COS is a potential probiotic by stimulating the growth of *Akkermansia*. However, *Akkermansia* may be sensitive to AOM and/or DSS, because it was not enriched in all of the AOM/DSS-treated groups, including the CACM, CACMe, CMCOS, and CMCOSe treatments.

Of particular interest, the abundance of *Lactobacillus*, which is most known for its essential use in food fermentation and as a probiotic, was significantly higher in the CACM and CACMe groups than in the other groups. The qPCR revealed that the abundance of *Lactobacillus* was significantly enriched in the CACM and CACMe groups at the 6th, 9th, and 10th weekends, consistent with the results of the LEfSe analysis of sequences in which *Lactobacillus* was one of the characteristic genera in the CACMe group. Even though *Lactobacillus* is only a minor member of the human colonic microbiota, the proportion of this genus is frequently either positively or negatively correlated with human disease and chronic conditions (Heeney et al., 2018). Many studies suggested that *Lactobacillus* was beneficial to intestinal health, whereas those of other studies indicated that *Lactobacillus* was enriched during disease, such as Crohn’s disease (Wang et al., 2014; Lewis et al., 2015), Type 2 diabetes (Karlsson et al., 2013), and head and neck squamous cell cancer (Guerrero-Preston et al., 2016). In this study, *L. johnsonii* was strongly positively correlated with the development of CRC. Although many studies reported that *L. johnsonii* was the most studied probiotic bacteria strain (Ashraf and Shah, 2014); Perez-Muñoz et al., 2014) found that spontaneous inflammation of the colons of core 1-derived *O*-glycans-deficient mice was associated with an increase of *L. johnsonii*. Thus, it is difficult to conclude whether *Lactobacillus* is a driver or just along for the ride in health and disease. Thus, the enrichment of *Lactobacillus* during the development of CRC in this study indicated that the role of *Lactobacillus* should be further investigated.

Little is known regarding the relationship between intestinal fungi and CRC. In this study, fungal diversity significantly decreased and fungal community composition notably shifted in the CACM group. These results are consistent with those of a previous study in which fecal samples were collected from 131 CRC patients and a distinct fungal dysbiosis and alteration of the fungal network was shown to potentially play important roles in polyp and CRC pathogenesis (Gao et al., 2017). In this study, treatment of COS recovered these changes in the CMCOS group. No significant difference was observed between the CK and CMCOS groups with respect to fungal diversity, and the samples from the CMCOS were clustered together with those from the CK group, and far away from those of the CACM group in the PCA analysis.

Interestingly, *Cladosporium* was a characteristic bacterial genus in the CMCOS group, and was one of the genera that strongly negatively correlated with tumor multiplicity, DAI and cytokine levels. Some *Cladosporium* metabolites have been reported to be effective at inhibiting the growth of human colon cancer cells. For instance, an isolated fungal taxol produced by *Cladosporium oxysporum* suppressed the growth of six different human pathogenic bacteria and the cancer cell line HCT15 (Gokul Raj et al., 2015). In addition, cladosporols that were purified and characterized as secondary metabolites from *Cladosporium tenuissimum* inhibited the growth of various human colon cancers derived cell lines (HT-29, SW480, and CaCo-2) in a time- and concentration-dependent manner, especially toward HT-29 cells (Zurlo et al., 2013). Thus, the COS-induced enrichment of *Cladosporium* may be a potential factor that inhibited CRC development. However, due to the very limited reports of fungi in CRC and the even less-reported effect of cage-exchanged on modulating mycobiota described in this study, the role of fungi in CRC should be pursued in future studies.

*Pichia kudriavzevii* has been isolated from human fecal samples (Madeeha et al., 2016) and has shown expressed efficient probiotic properties (Srinivas et al., 2017). Metabolites secreted by *P. kudriavzevii* AS-12 demonstrated anticancer effects by inhibiting cell proliferation and inducing intrinsic and extrinsic apoptosis in colon cancer cells (Saber et al., 2017). In this study, *P. kudriavzevii* was strongly negatively correlated with tumor multiplicity, disease activity index and cytokines. Thus, *P. kudriavzevii* may play an important role in preventing the development of CRC, and further research on *P. kudriavzevii* and CRC needs to be performed.

*Vagococcus fluvialis* was previously isolated and characterized as probiotic strain exhibiting a protective effect against vibriosis in sea bass (Sorroza et al., 2012) and was also detected in the root canals and midgut of *Culex quinquefasciatus* mosquitoes (Schirrmeister et al., 2009; Chandel et al., 2015). Román et al. (2013, 2015) reported that *V. fluvialis* and its extracellular products had a clear immunomodulatory activity *in vitro* as well as the ability to induce cytokine production related to the immune response. In the present study, *V. fluvialis* was highly positively correlated with cytokines, tumor multiplicity and disease activity index. A relationship between *V. fluvialis* and cytokines was easily observed, as transcripts of cytokines (IL-1, TNF-α, COX-2, and IL-10) were highly up-regulated in sea bass after a 1-h of incubation with the probiotic strain *V. fluvialis* L-21. However, this was the first time that relationship between *V. fluvialis* and CRC has been observed. Due to the limitation knowledge of related references, the role of *V. fluvialis* in CRC should be further studied.

In summary, the results of this study provided the first evidence that microbiota contributed to the antitumor activity of COS toward CRC. These results were supported by different presentations of CRC incidence, tumor multiplicity, expression of cytokines, bacterial community diversity and composition between the intervention groups, especially between the separated and cage-exchanged groups (CACM *vs.* CACMe, and CMCOS *vs.* CMCOSe). Notably, these data also provided evidence that COS inhibited CRC development by enriching probiotics (*Akkermansia* and butyrate-producing bacteria) and decreasing pathobionts (*Escherichia_Shigella* and *Enterococcus*). Moreover, this study also revealed that mycobiota was altered during the CRC development process, and COS also effectively recovered the intestinal fungal community composition. Thus, COS may be a potential therapeutic for the prevention of CRC development, in part through regulating the gut microbiota and mycobiota.

## Author Contributions

MW and GZ conceived and designed the experiments. JiaL, YA, PL, WX, and MW performed the experiments and collected the data. MW, JiaL, JinL, and DY analyzed the data. MW and GZ wrote the manuscript.

## Conflict of Interest Statement

The authors declare that the research was conducted in the absence of any commercial or financial relationships that could be construed as a potential conflict of interest.

## Footnotes

1 http://www.drive5.com/uparse/

2 http://www.arb-silva.de/

3 www.i-sanger.com

4 http://huttenhower.sph.harvard.edu/galaxy/
