# Supplementary material for: Chitooligosaccharides Prevents the Development of Colitis-Associated Colorectal Cancer by Modulating the Intestinal Microbiota and Mycobiota
Source: Front Microbiol. 2019 Sep 18;10:2101. doi: 10.3389/fmicb.2019.02101 (PMC6759605; doi:10.3389/fmicb.2019.02101)
Supplement: Supplementary file 1 [file Data_Sheet_1.docx]

**Figure S1. Representative images of hematoxylin and eosin (HE) staining of mouse colon tissue at the 10^th^ weekend.** Original magnification, 100× and 200×. CK: control treatment; COS: COS control treatment; CACM: AOM/DSS-induced colitis-associated CRC model mice; CMCOS, COS-treated CACM mice (300 mg/kg/d COS); CACMe, exchanged CACM treatment; CMCOSe: exchanged CMCOS treatment.


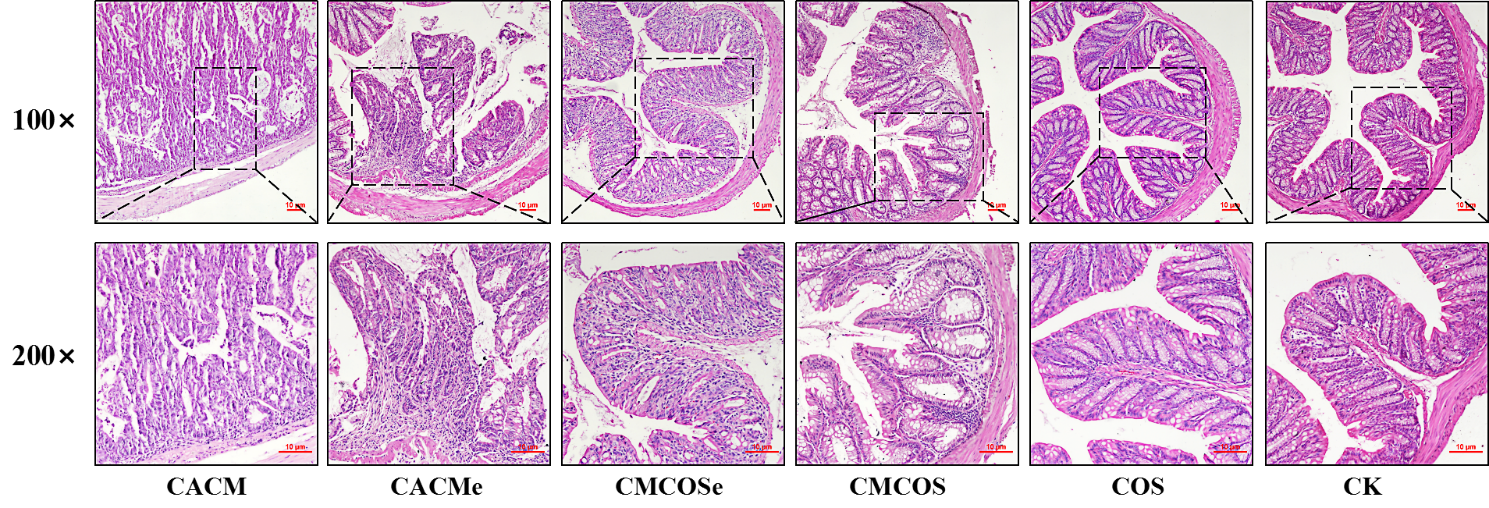


**Figure S2. The abundances of fecal total bacteria at the 0^th^, 1^st^, 3^rd^, 6^th^, 9^th^ and 10^th^ weekends.** The results were presented as the mean ± standard deviation (SD); *n* = 8 for each treatment. Analysis of variance (ANOVA) was used, significant differences (*P* < 0.05) between treatments are indicated by the letters a, b, or c. CK: control treatment; COS: COS control treatment; CACM: AOM/DSS-induced colitis-associated CRC model mice; CMCOS, COS-treated CACM mice (300 mg/kg/d COS); CACMe, exchanged CACM treatment; CMCOSe: exchanged CMCOS treatment.


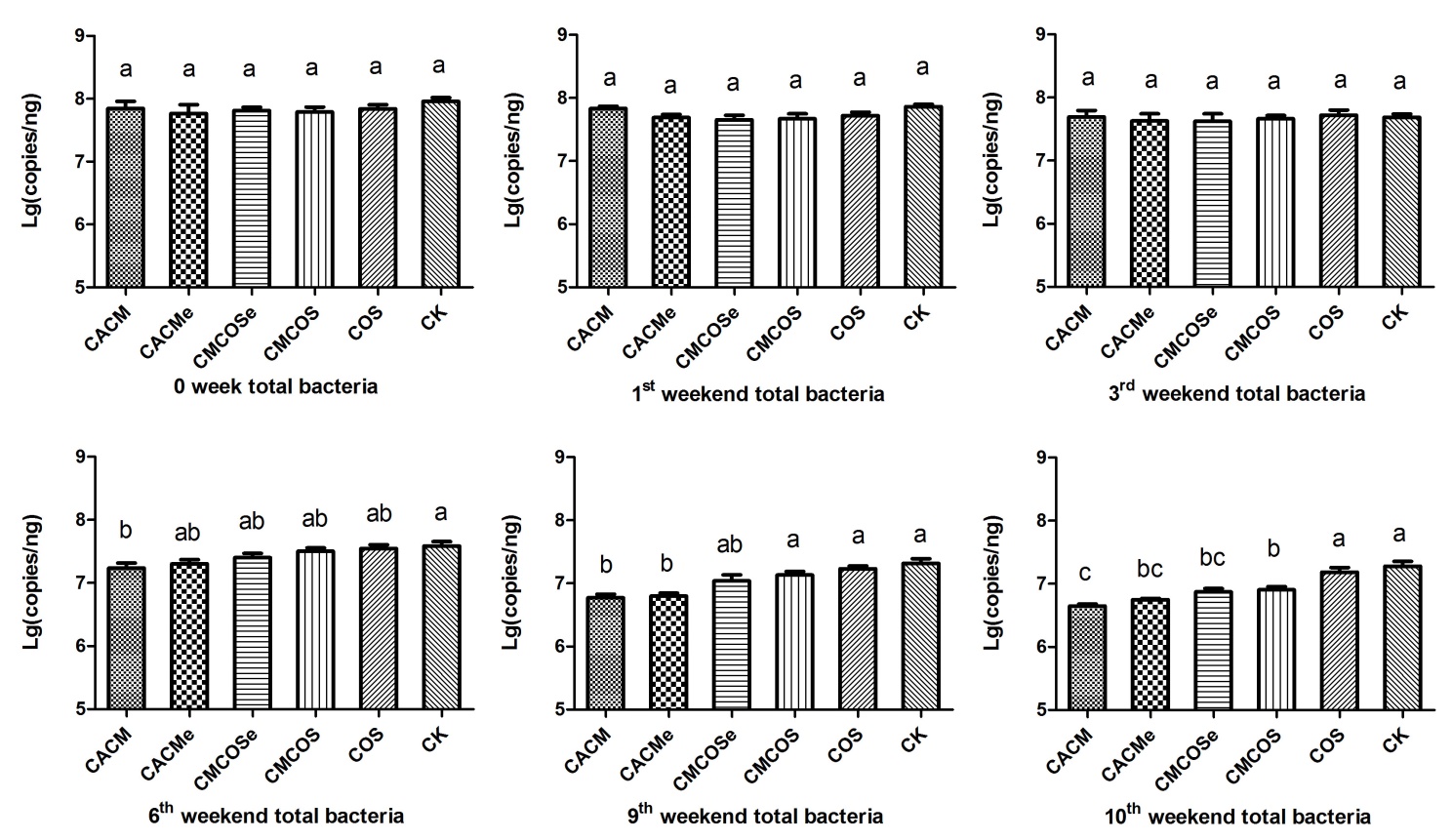


**Figure S3. The abundances of fecal *Fusobacterium nucleatum* at the 0^th^, 1^st^, 3^rd^, 6^th^, 9^th^ and 10^th^ weekends.** The results were presented as the mean ± standard deviation (SD); *n* = 8 for each treatment. Analysis of variance (ANOVA) was used, significant differences (*P* < 0.05) between treatments are indicated by the letters a, b, or c. CK: control treatment; COS: COS control treatment; CACM: AOM/DSS-induced colitis-associated CRC model mice; CMCOS, COS-treated CACM mice (300 mg/kg/d COS); CACMe, exchanged CACM treatment; CMCOSe: exchanged CMCOS treatment.


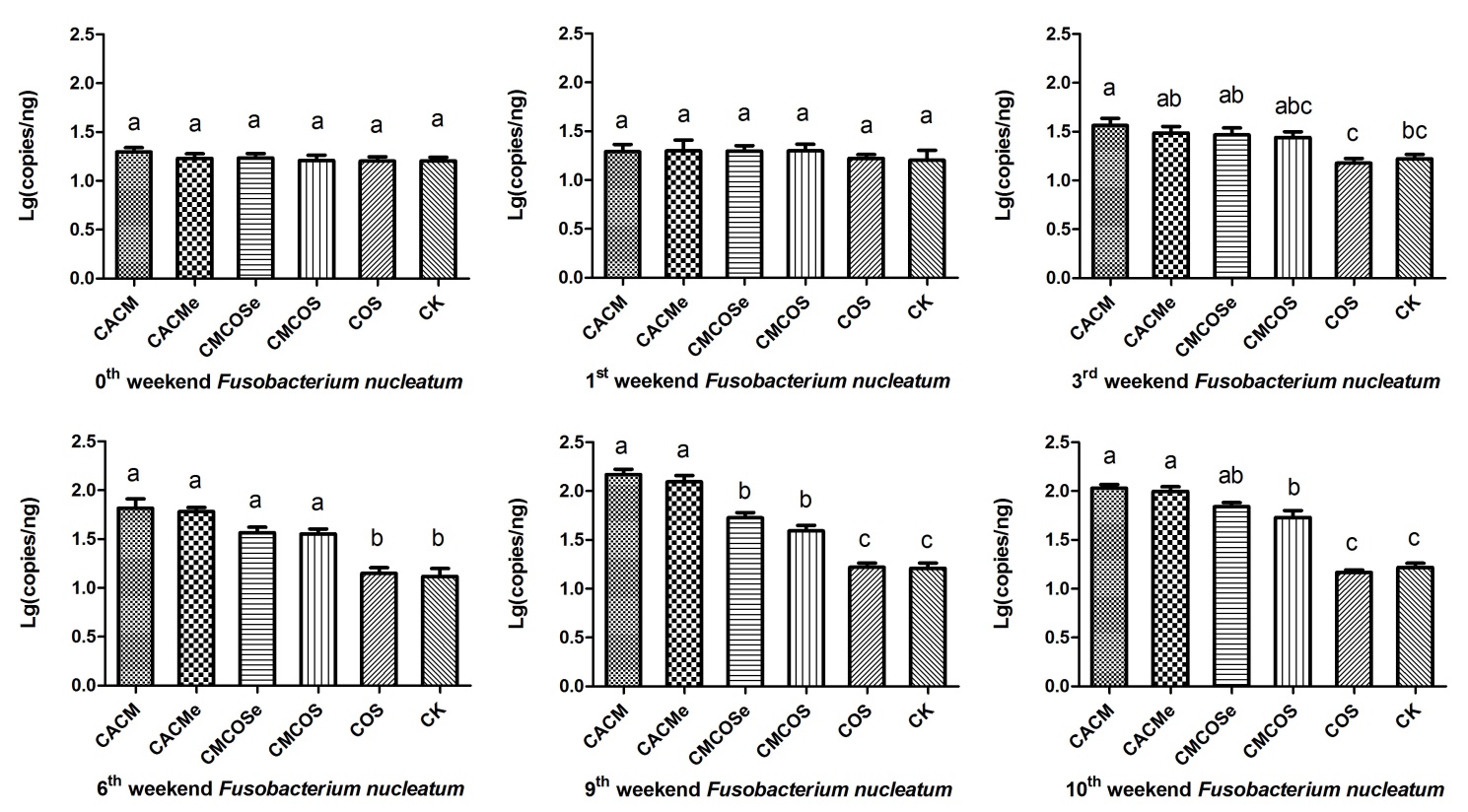


**Figure S4. The abundances of fecal *Enterococcus* at the 0^th^, 1^st^, 3^rd^, 6^th^, 9^th^ and 10^th^ weekends.** The results were presented as the mean ± standard deviation (SD); *n* = 8 for each treatment. Analysis of variance (ANOVA) was used, significant differences (*P* < 0.05) between treatments are indicated by the letters a, b, c, or d. CK: control treatment; COS: COS control treatment; CACM: AOM/DSS-induced colitis-associated CRC model mice; CMCOS, COS-treated CACM mice (300 mg/kg/d COS); CACMe, exchanged CACM treatment; CMCOSe: exchanged CMCOS treatment.


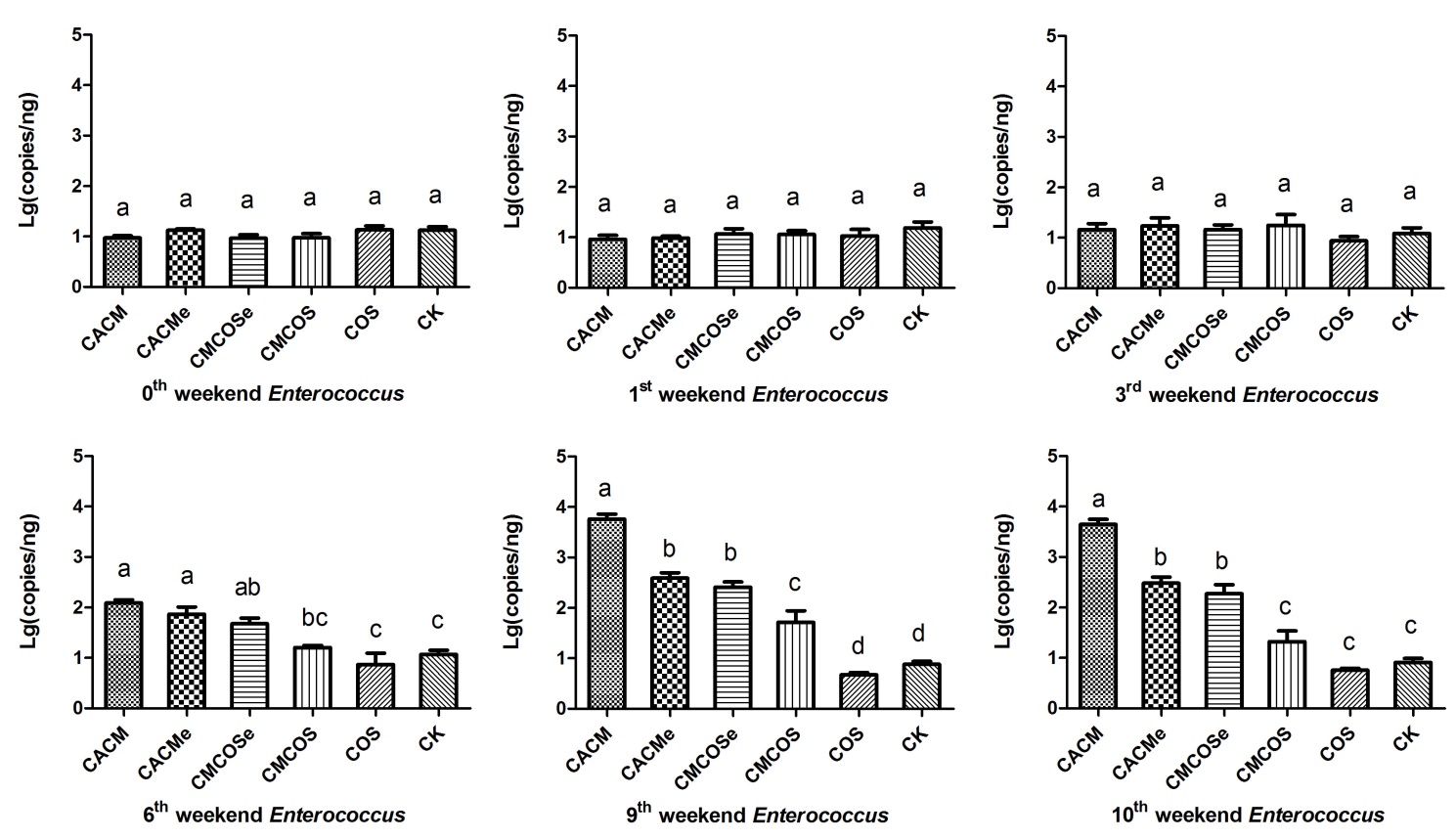


**Figure S5. The abundances of fecal *Lactobacillus* at the 0^th^, 1^st^, 3^rd^, 6^th^, 9^th^ and 10^th^ weekends.** The results were presented as the mean ± standard deviation (SD); *n* = 8 for each treatment. Analysis of variance (ANOVA) was used, significant differences (*P* < 0.05) between treatments are indicated by the letters a, b, or c. CK: control treatment; COS: COS control treatment; CACM: AOM/DSS-induced colitis-associated CRC model mice; CMCOS, COS-treated CACM mice (300 mg/kg/d COS); CACMe, exchanged CACM treatment; CMCOSe: exchanged CMCOS treatment.


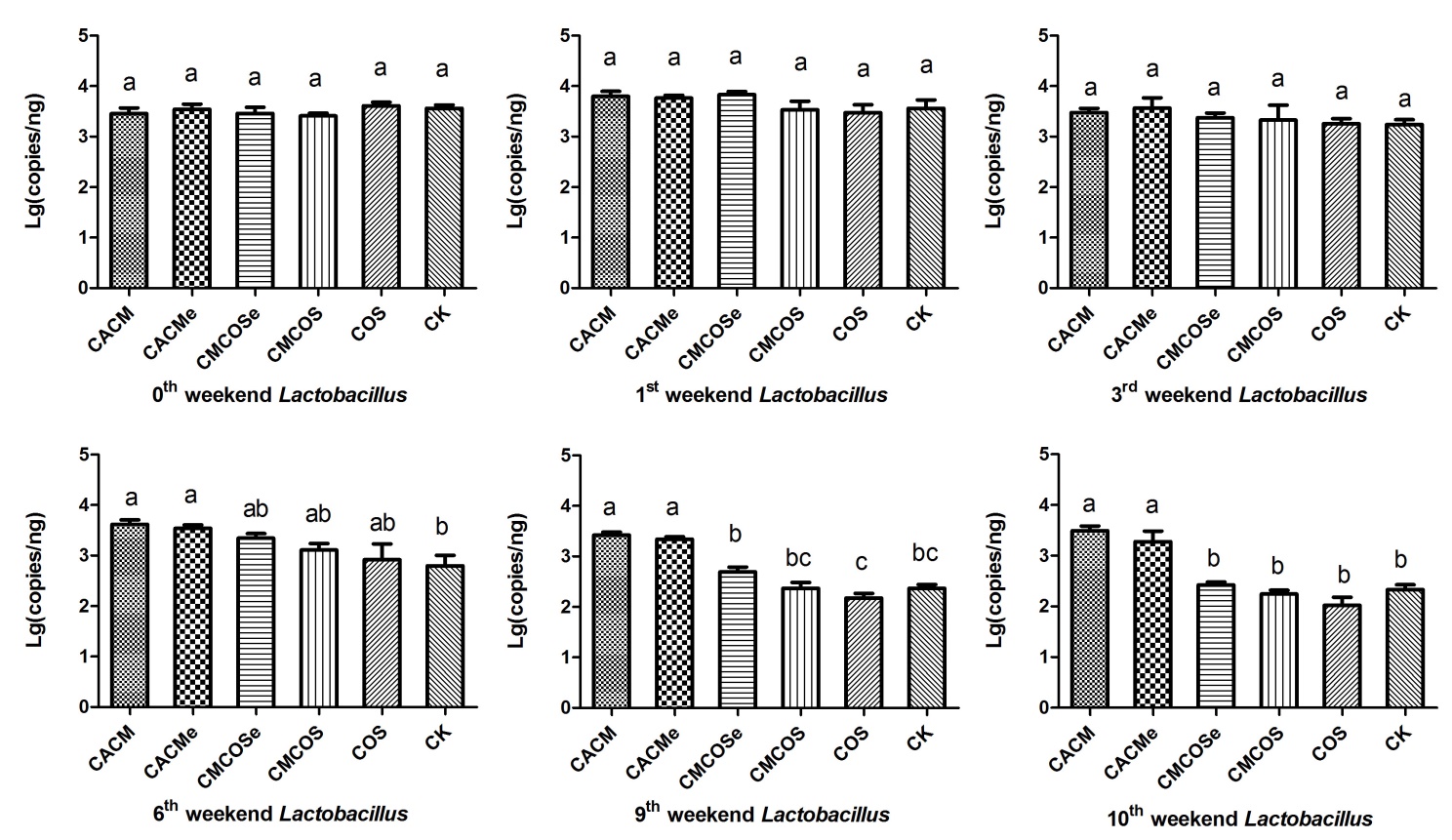


**Figure S6. The abundances of fecal butyrate-producing bacteria at the 0^th^, 1^st^, 3^rd^, 6^th^, 9^th^ and 10^th^ weekends.** The results were presented as the mean ± standard deviation (SD); *n* = 8 for each treatment. Analysis of variance (ANOVA) was used, significant differences (*P* < 0.05) between treatments were indicated by the letters a, b, c, or d. CK: control treatment; COS: COS control treatment; CACM: AOM/DSS-induced colitis-associated CRC model mice; CMCOS, COS-treated CACM mice (300 mg/kg/d COS); CACMe, exchanged CACM treatment; CMCOSe: exchanged CMCOS treatment.

**
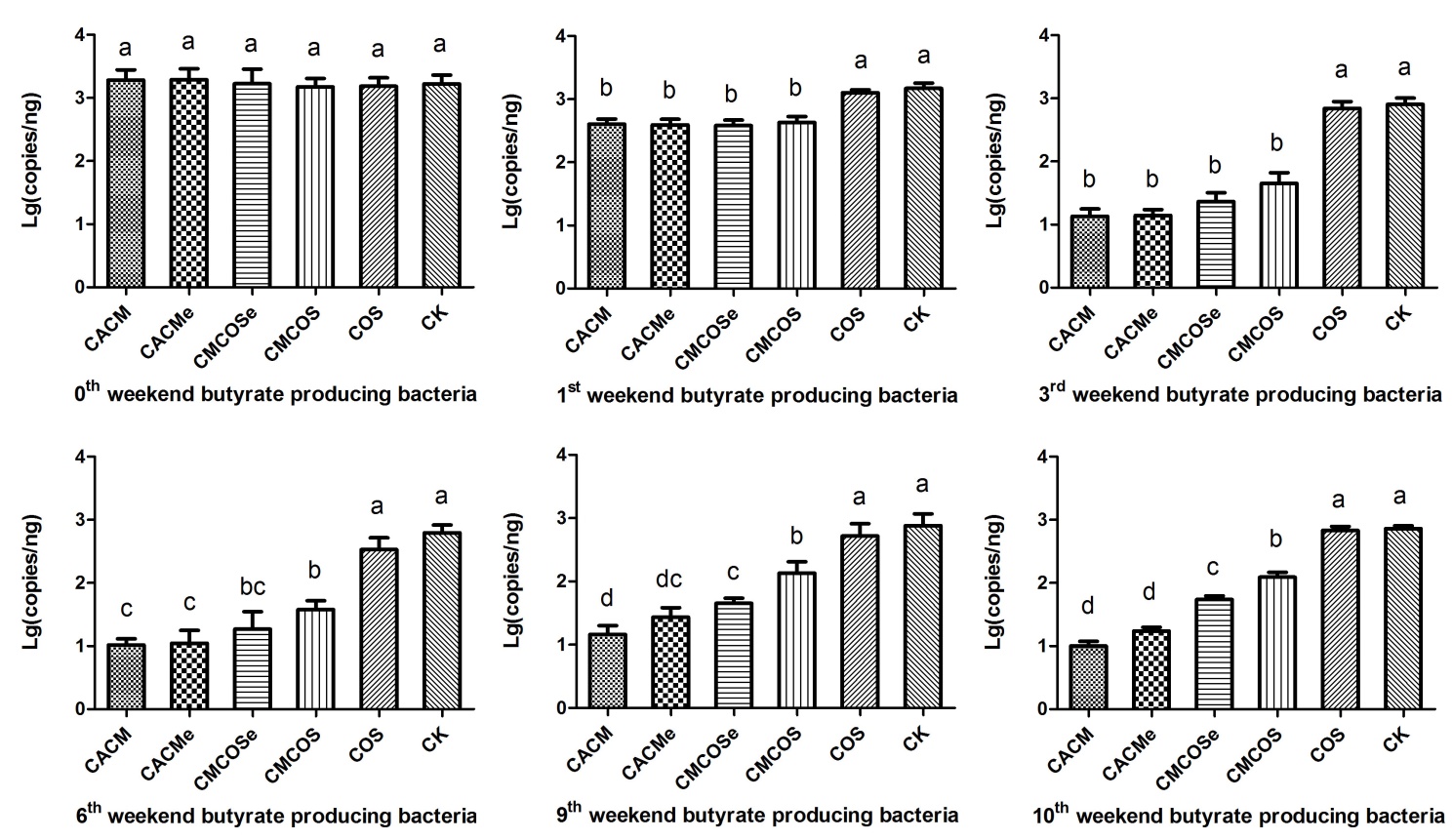
**

**Figure S7. Analysis of bacterial similarities at different categorical levels.** The statistics were performed based on bray-curtis distance, *n* = 4. CK: control treatment; COS: COS control treatment; CACM: AOM/DSS-induced colitis-associated CRC model mice; CMCOS, COS-treated CACM mice (300 mg/kg/d COS); CACMe, exchanged CACM treatment; CMCOSe: exchanged CMCOS treatment.


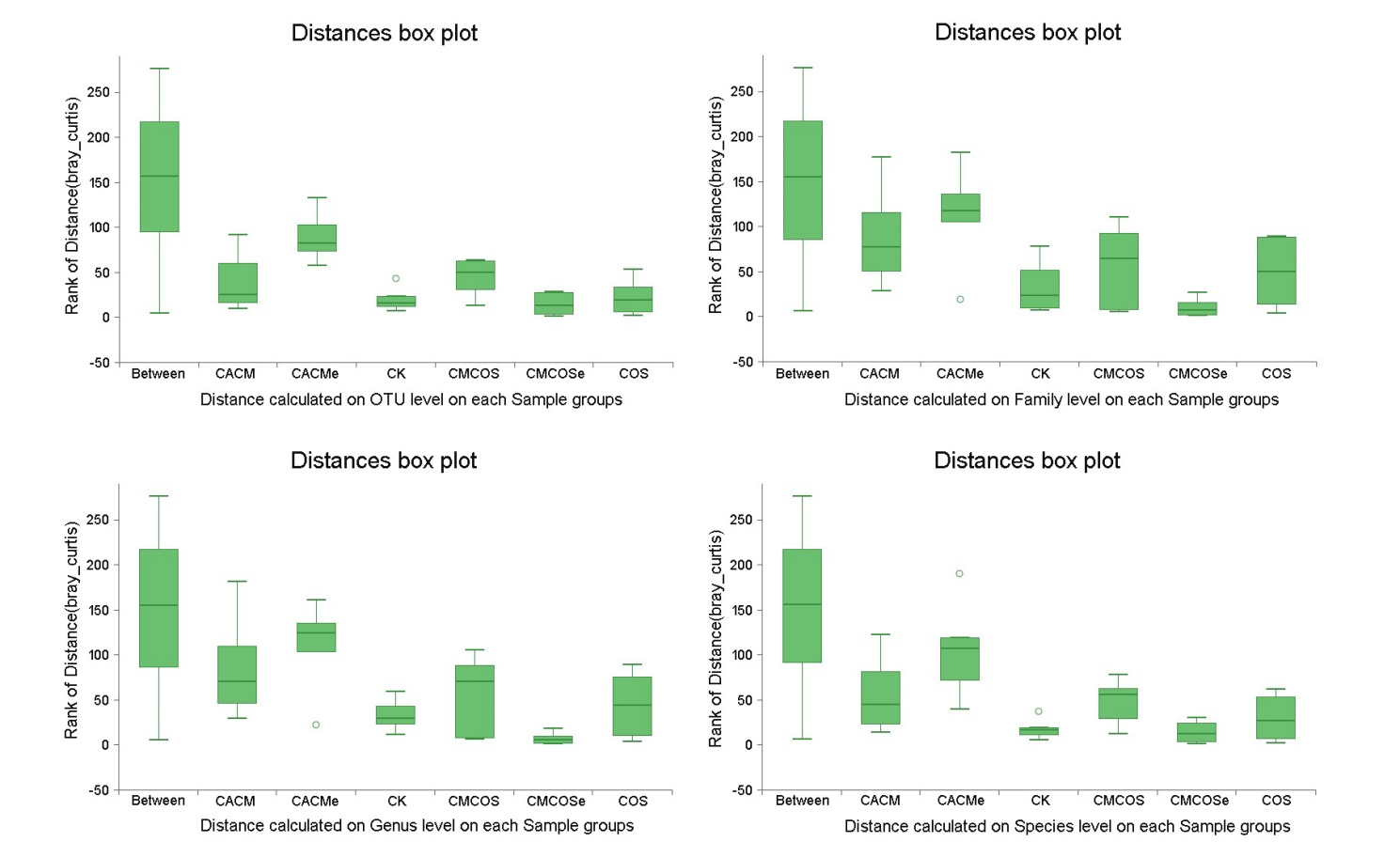


**Figure S8. Pincipal components analysis (PCA) of 18S rRNA gene-sequencing.** PC1, PC2 and PC3 explained 39.06, 17.48 and 15.62% of variation, respectively. The statistics were performed based on bray-curtis distance, *n* = 4. CK: control treatment; COS: COS control treatment; CACM: AOM/DSS-induced colitis-associated CRC model mice; CMCOS, COS-treated CACM mice (300 mg/kg/d COS); CACMe, exchanged CACM treatment; CMCOSe: exchanged CMCOS treatment.


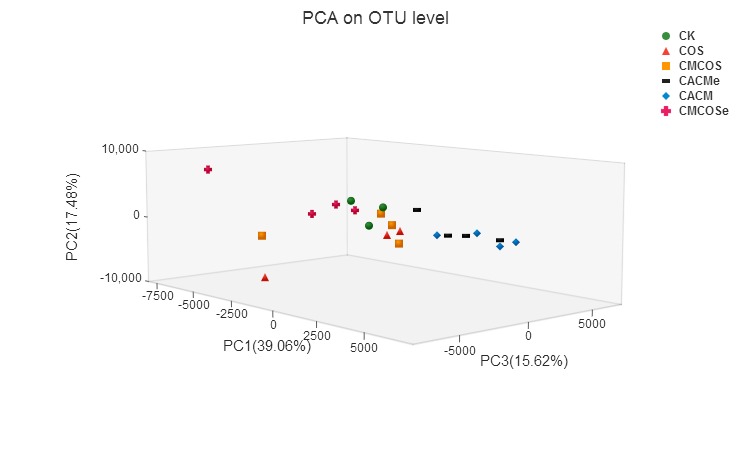


**Figure S9.** **Relative abundances at the phylum, family and genus levels for fungi that exceeded 1% of the total.** The results are presented as the mean ± standard deviation (SD); *n* = 3 for CK and COS, *n* = 4 for CACM, CMCOS, CACMe and CMCOSe. * Analysis of variance (ANOVA) was used, significant differences between CK and treatments were indicated (*P* < 0.05). CK: control treatment; COS: COS control treatment; CACM: AOM/DSS-induced colitis-associated CRC model mice; CMCOS, COS-treated CACM mice (300 mg/kg/d COS); CACMe, exchanged CACM treatment; CMCOSe: exchanged CMCOS treatment.


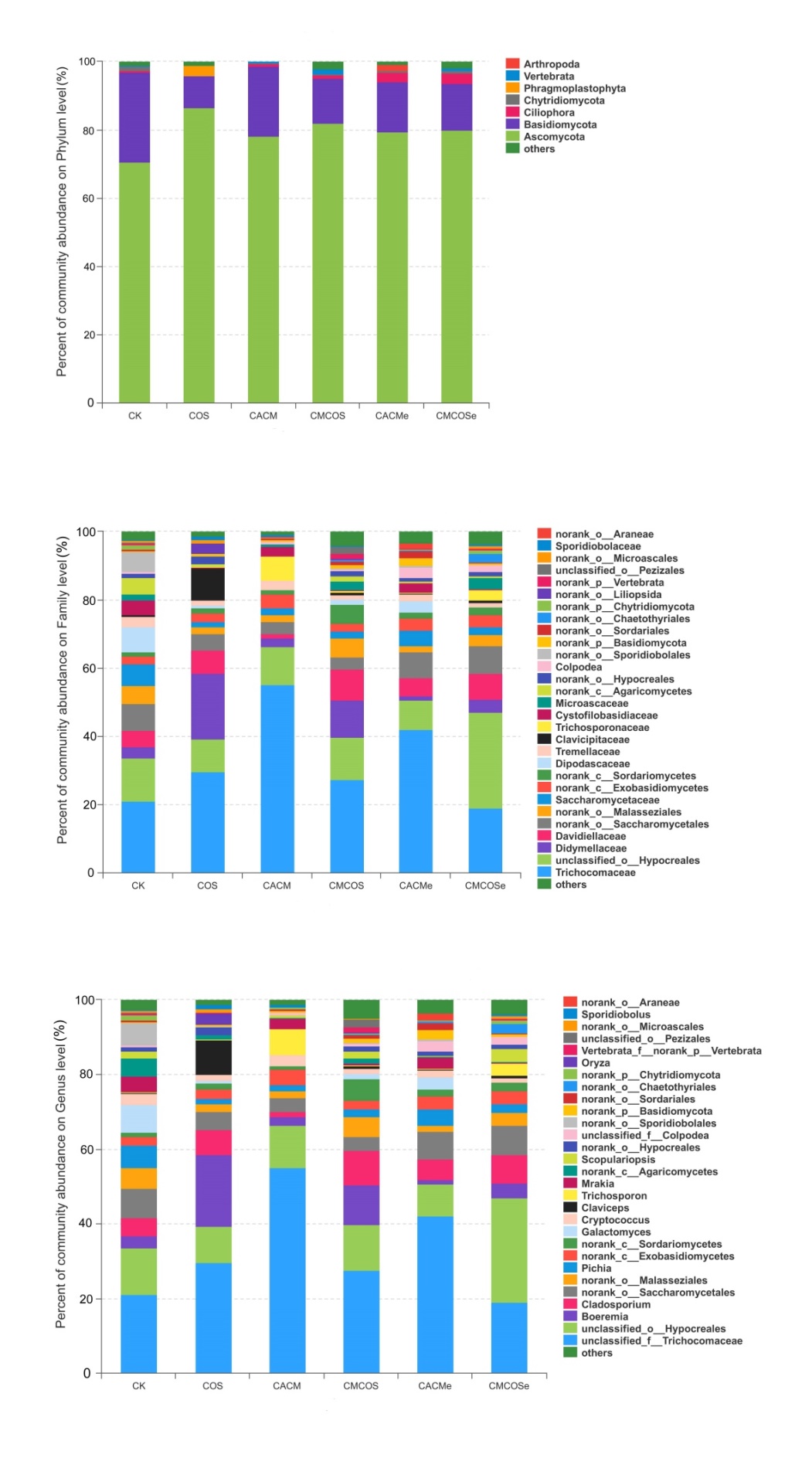


**Figure S10. The correlation between microbiota at species level and environmental factors (DAI, tumor multiplicity and cytokines).** The most abundant 50 species in each sample were used to perform the hierarchical clustering and heatmap analyses based on Spearman correlation coefficient. *n* = 4. TUM: tumor multiplicity; DAI: disease activity index.

**
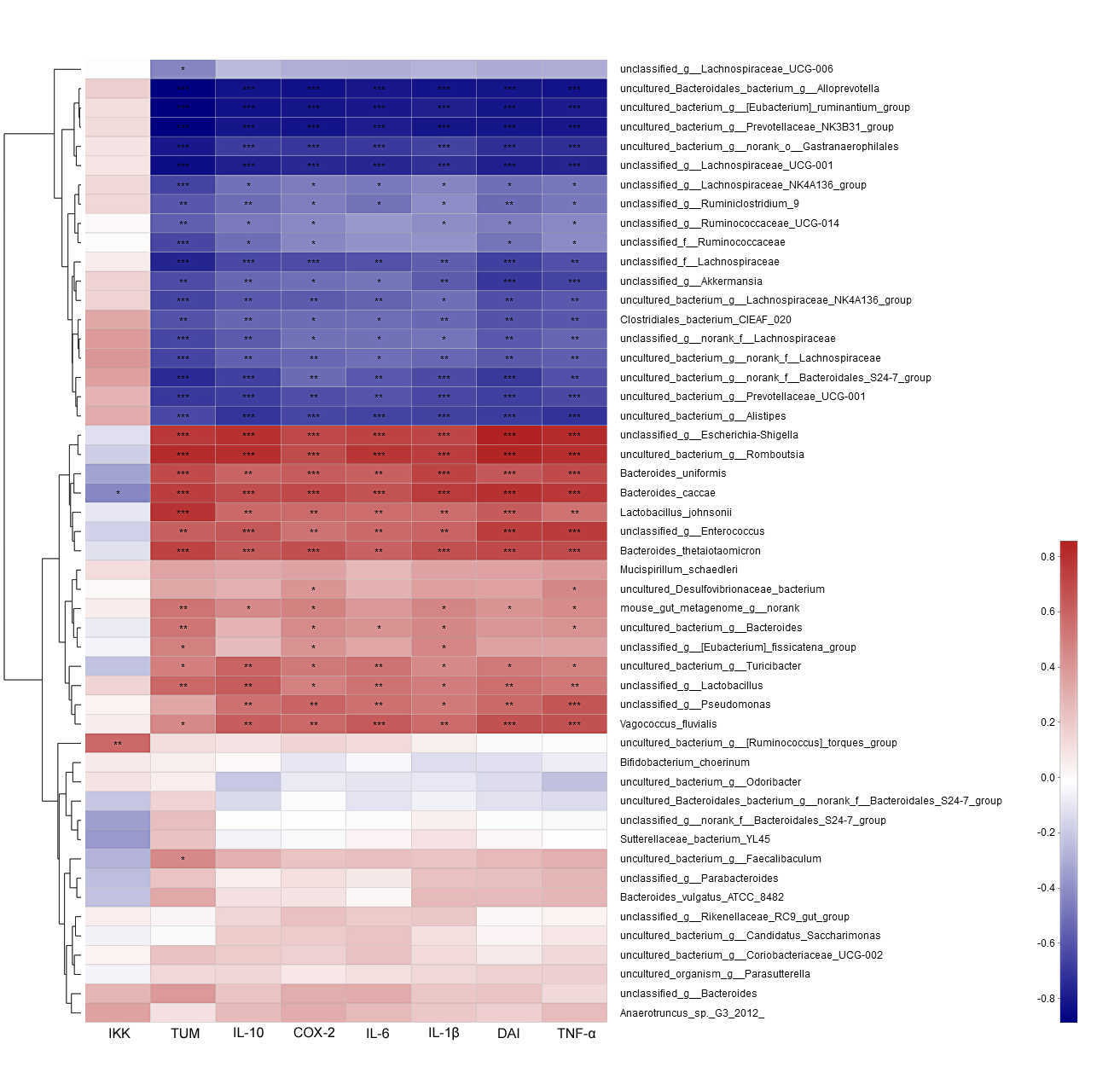
**

**Figure S11. The correlation between mycobiota at species level and environmental factors (DAI, tumor multiplicity and cytokines).** The most abundant 50 species in each sample were used to perform the hierarchical clustering and heatmap analyses based on Spearman correlation coefficient. *n* = 4. TUM: tumor multiplicity; DAI: disease activity index.


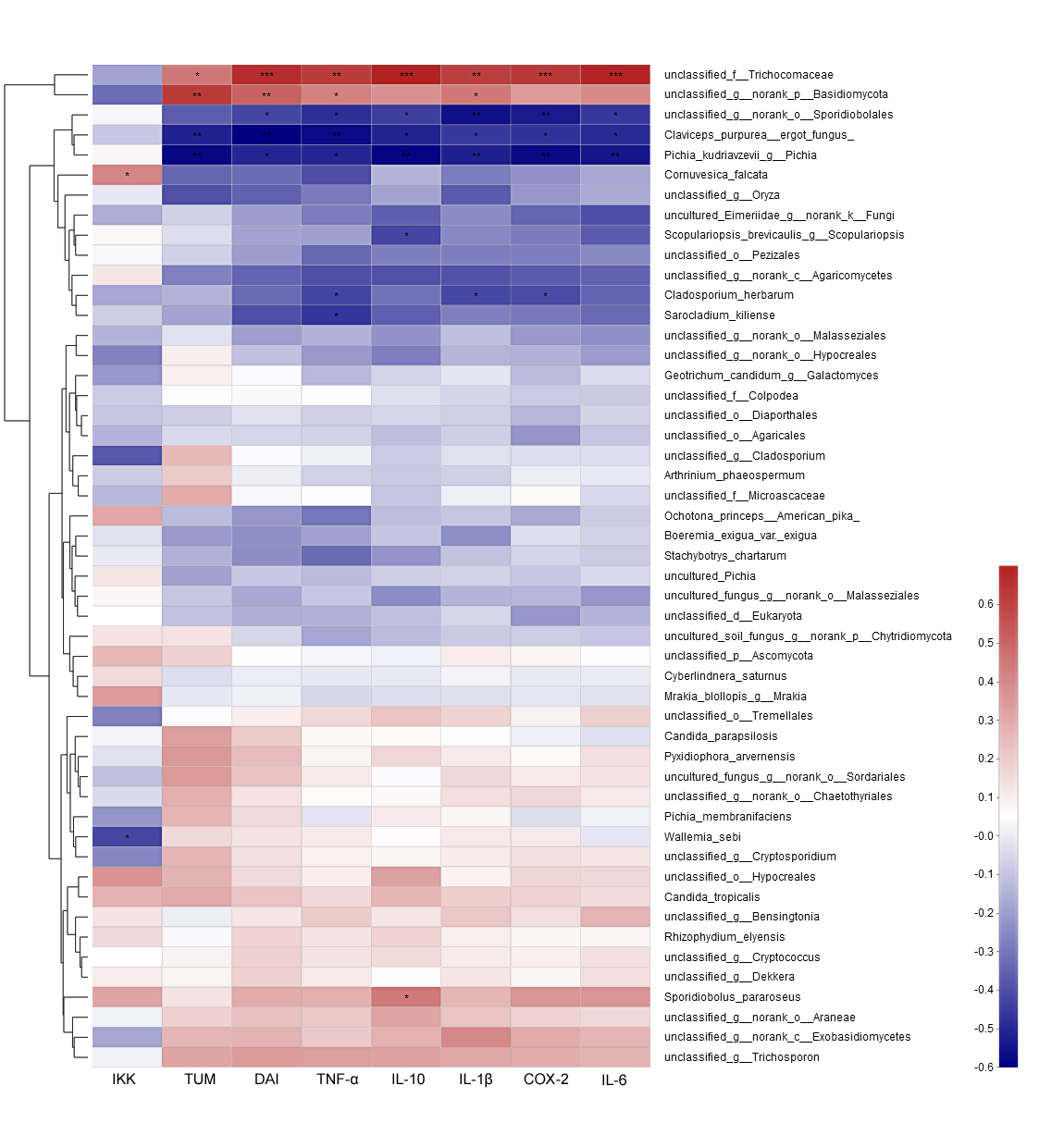


**Table S1. The primer sequences for specific bacteria by real-time PCR**

| **Traget bacterial group** | **Primer name** | **Primer sequences** | **R^2^** | **Amplification efficiency (%)** |
| --- | --- | --- | --- | --- |
| Total bacteria ^[1]^ | 341F | CCTACGGGAGGCAGCAG | 0.998 | 105.8 |
|  | 518R | ATTACCGCGGCTGCTGG |  |  |
| *Lactobacillus* ^[2]^ | Lac-F | AGCAGTAGGGAATCTTCCA | 0.993 | 99.3 |
|  | Lac-R | CACCGCTACACATGGAG |  |  |
| *Enterococcus* ^[3]^ | Ent-F | CCCTTATTGTTAGTTGCCATCATT | 0.996 | 103.6 |
|  | Ent-R | ACTCGTTGTACTTCCCATTGT |  |  |
| *Fusobacterium nucleatum* ^[4]^ | Fus-F | CAACCATTACTTTAACTCTACCATGTTCA | 0.993 | 92.9 |
|  | Fus-R | GTTGACTTTACAGAAGGAGATTATGTAAAAATC |  |  |
| Butyrate producing bacteria ^[5]^ | Bco-F | GCIGAICATTTCACITGGAAYWSITGGCAYATG | 0.994 | 95.2 |
|  | Bco-R | CCTGCCTTTGCAATRTCIACRAANGC |  |  |

Note: The PCR amplification procedures were: 95°C 30 s; 40 cycles of denaturing at 95°C for 5 s, and annealing/extension at 60°C for 30 s. After all cycles, dissociation curves were performed as 95°C for 15s, 60°C for 1 min, and 95°C for 15 s. The references were present as follows.

[1] Muyzer, G, De Waal EC, Uitterlinden AG. Profiling of complex microbial populations by denaturing gradient gel electrophoresis analysis of polymerase chain reaction-amplified gene coding for 16S rRNA. *Appl Environ Microbiol* 1993; **59**: 695-700.

[2] Collado MC, Isolauri E, Laitinen K, et al. Distinct composition of gut microbiota during pregnancy in overweight and normal-weight women. *Am J Clini Nutri* 2008; **88**: 894-899.

[3] Rinttilä T, Kassinen A, Malinen E, et al. Development of an extensive set of 16S rDNA-targeted primers for quantification of pathogenic and indigenous bacteria in faecal samples by real-time PCR. *J Appl Microbiol* 2004; 97(6): 1166-1177.

[4] Castellarin, M., Warren, R., Freeman, J., Dreolini, L., Krzywinski, M., Strauss, J. et al. Fusobacterium nucleatum infection is prevalent in human colorectal carcinoma. *Genome Res* 2012; **22**: 299–306.

[5] Louis P, Flint HJ. Development of a semiquantitative degenerate real-time pcr-based assay for estimation of numbers of butyryl-coenzyme A (CoA) CoA transferase genes in complex bacterial samples. *Appli Environ Microbiol*, 2007; **73**(6): 2009-2012.

**Table S2. The dynamic changes of body weights in the experimental period.**

| **Time (week)** | **CK** | **COS** | **CACM** | **CMCOS** | **CACMe** | **CMCOSe** |
| --- | --- | --- | --- | --- | --- | --- |
| 0 | 23.76±1.62 | 23.59±1.28 | 23.92±1.06 | 23.41±0.51 | 23.76±0.95 | 23.59±1.01 |
| 1 | 23.85±1.11 | 24.70±1.12 | 23.18±1.27 | 22.73±1.08 | 22.72±0.82 | 22.73±0.67 |
| **2** | **24.13±0.91^a^** | **24.79±1.57^a^** | **21.01±1.01^b^** | **20.94±0.61^b^** | **20.76±0.52^b^** | **20.84±0.418^b^** |
| 3 | 24.72±1.46^a^ | 24.63±1.29^a^ | 22.03±1.50^b^ | 21.80±1.23^b^ | 21.73±0.26^b^ | 21.99±0.69^b^ |
| 4 | 23.58±1.11^ab^ | 24.26±1.53^a^ | 22.93±1.74^ab^ | 23.13±0.81^ab^ | 22.52±0.58^ab^ | 23.41±0.78^b^ |
| **5** | **24.13±1.23^a^** | **24.94±1.59^a^** | **20.13±1.39^b^** | **21.85±1.01^b^** | **21.41±1.42^b^** | **21.18±1.16^b^** |
| 6 | 24.80±1.15^ab^ | 25.50±1.17^a^ | 22.32±1.72^c^ | 23.51±1.06^abc^ | 22.68±1.19^bc^ | 22.66±2.28^bc^ |
| 7 | 25.40±1.29^ab^ | 26.21±1.28^a^ | 23.44±1.48^b^ | 24.81±1.20^ab^ | 24.15±1.02^ab^ | 23.83±2.01^b^ |
| **8** | **26.42±0.76^a^** | **27.11±1.56^a^** | **19.34±1.01^c^** | **21.70±1.30^b^** | **19.96±1.27^bc^** | **21.02±1.61^bc^** |
| 9 | 26.85±0.87^a^ | 27.51±1.57^a^ | 21.29±0.37^d^ | 23.93±0.58^b^ | 21.75±1.11^cd^ | 23.30±0.91^bc^ |
| 10 | 27.14±1.19^a^ | 27.68±1.86^a^ | 22.01±0.41^d^ | 25.34±1.03^b^ | 22.95±0.57^cd^ | 24.43±0.81bc |

Note: The results were presented as the mean ± standard deviation (SD); n = 8 for each treatment. Analysis of variance (ANOVA) was used, significant differences (*P* < 0.05) between treatments are indicated by the letters a, b, or c. CK, control group; COS, COS treated group; CACM, AOM/DSS induced colitis-associated CRC model mice; CMCOS, COS treated CACM mice (300 mg/kg COS), CACMe, exchanged CACM group; CMCOSe: exchanged CMCOS group.

**Table S3. The dynamic changes of disease activity index in the experimental period.**

| **Time (week)** | **CK** | **COS** | **CACM** | **CMCOS** | **CACMe** | **CMCOSe** |
| --- | --- | --- | --- | --- | --- | --- |
| 0 | 0.00±0.00 | 0.00±0.00 | 0.00±0.00 | 0.00±0.00 | 0.00±0.00 | 0.00±0.00 |
| 1 | 0.00±0.00^b^ | 0.00±0.00^b^ | 0.45±0.25^a^ | 0.25±0.30^ab^ | 0.54±0.25^a^ | 0.29±0.21^ab^ |
| **2** | **0.00±0.00^b^** | **0.00±0.00^b^** | **1.83±0.31^a^** | **1.59±0.16^a^** | **1.75±0.15^a^** | **1.71±0.21^a^** |
| 3 | 0.00±0.00^b^ | 0.00±0.00^b^ | 1.46±0.25^a^ | 1.21±0.25^a^ | 1.37±0.28^a^ | 1.25±0.30^a^ |
| 4 | 0.00±0.00^b^ | 0.00±0.00^b^ | 0.92±0.46^a^ | 0.50±0.18^a^ | 0.62±0.45^a^ | 0.58±0.23^a^ |
| **5** | **0.00±0.00^d^** | **0.00±0.00^d^** | **2.79±0.25^a^** | **2.00±0.40^c^** | **2.50±0.31^ab^** | **2.08±0.46^bc^** |
| 6 | 0.00±0.00^d^ | 0.00±0.00^d^ | 2.19±0.26^a^ | 1.08±0.30^c^ | 2.04±0.37^ab^ | 1.29±0.70^c^ |
| 7 | 0.00±0.00^d^ | 0.00±0.00^d^ | 1.52±0.26^a^ | 0.67±0.18^c^ | 1.33±0.44^ab^ | 0.96±0.41^bc^ |
| **8** | **0.00±0.00^d^** | **0.00±0.00^d^** | **3.95±0.12^a^** | **2.75±0.35^c^** | **3.58±0.46^ab^** | **3.13±0.25^c^** |
| 9 | 0.00±0.00^d^ | 0.00±0.00^d^ | 3.22±0.27^a^ | 2.19±0.18^c^ | 2.72±0.25^b^ | 2.33±0.27^c^ |
| 10 | 0.00±0.00^d^ | 0.00±0.00^d^ | 2.80±0.38^a^ | 1.57±0.25^c^ | 2.45±0.27^ab^ | 1.76±0.42^c^ |

Note: The results were presented as the mean ± standard deviation (SD); *n* = 8 for each treatment. Analysis of variance (ANOVA) was used, significant differences (*P* < 0.05) between treatments are indicated by the letters a, b, or c. CK, control group; COS, COS treated group; CACM, AOM/DSS-induced colitis-associated CRC model mice; CMCOS, COS-treated CACM mice (300 mg/kg COS), CACMe, exchanged CACM group; CMCOSe: exchanged CMCOS group.

**Table S4. The histological assessment of each mouse in each group**

|  | CACM | CACMe | CMCOSe | CMCOS | COS | CK |
| --- | --- | --- | --- | --- | --- | --- |
| Normal tissue  Colitis  Low-grade intraepithelial neoplasia  High-grade intraepithelial neoplasia  Adenocarcinoma | 0  0  0  6  2 | 0  0  1  6  1 | 0  1  4  3  0 | 0  2  5  1  0 | 8  0  0  0  0 | 8  0  0  0  0 |

*n =* 8 for each group.

The histological assessment was based on dysplasia and intestinal cancer classification as previously reported ^[1-2]^.

1. Riddell RH, Goldman H, Ransohoff DF, et al. Dysplasia in inflammatory bowel disease: standardized classification with provisional clinical applications. *Hum Pathol* 1983;**14**(11):931-68.
2. Boivin GP 1, Washington K, Yang K, et al. Pathology of Mouse Models of Intestinal Cancer: Consensus Report and Recommendations. *Gastroenterology*. 2003;**124**(3):762-77.
